# Development of the First Health-Related Quality of Life Questionnaires in Arabic for Women with Polycystic Ovary Syndrome (Part I): The Creation and Reliability Analysis of PCOSQoL-47 and PCOSQoL-42 Questionnaires

**DOI:** 10.7759/cureus.14735

**Published:** 2021-04-28

**Authors:** Samih A Odhaib, Fatemeh Nasiri Amiri, Mahmood T Altemimi, Husam J Imran, Haider A Alidrisi, Miaad J Mohammed, Abbas A Mansour

**Affiliations:** 1 Adult Endocrinology, Faiha Specialized Diabetes, Endocrine and Metabolism Center (FDEMC) College of Medicine, University of Basrah, Basrah, IRQ; 2 Obstetrics and Gynecology, Health Research Institute, Infertility and Health Reproductive Research Center, Babol University of Medical Sciences, Babol, IRN; 3 Diagnostic Radiology, Al-Refaee General Hospital, Thi-Qar Health Directorate, Thi-Qar, IRQ

**Keywords:** content analysis, face validity, pcos, psychometric, qualitative research, quality of life, questionnaire, sexuality, test-retest

## Abstract

Background and objective

We lack a reliable and validated health-related quality of life (HRQoL) questionnaire to measure the negative impact of polycystic ovary syndrome (PCOS) on the various aspects of the lives of Arabic women, which addresses sexuality in married women only. Hence, we aimed to develop two separate, simple, reliable, validated, and easily interpretable HRQoL questionnaires in Arabic for married and unmarried women with PCOS for effective QoL evaluation.

Materials and methods

The development and reliability analysis of the Polycystic Ovary Syndrome Quality of Life (PCOSQoL)-47 and PCOSQoL-42 involved two quantitative and qualitative phases. Phase 1 included retrieval of 158 items from 584 PubMed articles, item reduction, Arabic translation, content and face validity testing, creation of a five-domain draft (53 items for married and 45 items for unmarried women), with no sexuality domain for unmarried women. Phase 2 involved test-retest reliability, which involved using the Spearman's correlation, Wilcoxon nonparametric signed-rank, and internal consistency using Cronbach's-alpha, inter-item, and intraclass correlation (ICC) coefficients, as well as creating a second draft (47 items for married and 42 items for unmarried women).

Results

The content validity indices testing by 26 healthcare experts decreased the item pool to 57 items for married and 45 items for unmarried women. Face validity by another 30 experts and 30 women from each group resulted in a further reduction to 53 items for married and 45 items for unmarried women, to be tested in a pilot study, which included another 30 women from each group. Test-retest reliability analysis by 195 married and 173 unmarried women revealed significantly excellent redundancy, reliability, and stability of items (highly significant Cronbach's alpha and ICC by internal consistency testing), and reduced the item pool to 47 items for married and 42 items for unmarried women.

Conclusions

Both questionnaires were found to be highly reliable for the HRQoL evaluation among both married and unmarried Arabic women with different phenotypes of PCOS.

## Introduction

The phenotypic heterogeneity in women with polycystic ovary syndrome (PCOS) is broad and associated with significant adverse effects on their health-related quality of life (HRQoL) [1]. These effects often manifest as psychological and physical distress, and self-esteem misperception, due to the failure to conform to the idealized norms of women's aesthetic and optimal health standards [2].

The symptoms perception related to PCOS is often influenced and characterized by complex cultural systems of beliefs, values, and ideals of particular societies/communities [3]. Many authors have translated the HRQoL questionnaires containing domains or items pertaining to individual sexuality to evaluate their populations [3-7], but they often fail to take into account the local community norms regarding sexual relationships in specific cultures, where sex may be confined to marriage and marital sex is the only legitimate sexual relationship allowed. According to Quranic instructions, any sexual relationships outside of marriage are frowned upon, and this view is zealously held in almost all predominantly Muslim communities. Consequently, many women in such communities are quite reluctant to discuss sexuality publicly. They seem to place great value on modesty and privacy and hold the view that questions related to sexuality are socially unacceptable [8], and such attitudes often prompt many women to be hesitant to respond to questionnaires that contain such queries and make the comparison between HRQoLs unreliable to some degree.

In view of this, we tried to develop two simple, reliable, validated, consistent, interpretable, and self-administered HRQoL questionnaires in Arabic, which were individualized for married and unmarried women with PCOS, to be used as an effective tool in assessing the HRQoL in these women, in order to facilitate further studies in this area among Arabic-speaking populations.

## Materials and methods

This study passed three phases to evolve into the final drafts of the PCOSQoL-42 and PCOSQoL-47. Phase 3, which included the assessment of the validation of the two questionnaires, will be discussed in a separate paper.

Phase 1: Questionnaire items retrieval (November 2018-March 2019)

We reviewed the PubMed database for the terms "polycystic" and "PCOS", with "quality of life" in the article titles, abstracts, and texts, among articles published till 2019 in the English language. We found 274 articles with these search terms. Another 310 articles were retrieved from the references sections of the original search articles after excluding duplications.

The authors reviewed these 584 articles to retrieve 158 possible HRQoL complaint-related items among women in different life domains. The authors added additional 32 items from their daily practice. These 190 items were reviewed for duplication and consistency through an item-reduction phase to be more representative and concise. This phase reduced the items to 82 items, which were agreed upon by the authors. A second item-reduction phase was performed to create an initial draft with a subset of a 67-item questionnaire for married women and a subset of a 52-item questionnaire for unmarried women with PCOS.

Translation of the Questionnaire Items to Arabic

The translation was done through two stages: the first stage of forwarding translation involved translating the items from the mother's native language (English) to the target (Arabic) language by three independent endocrinologists. The authors agreed that the final draft of the translation should be in a simple common language, with minimal use of the medical terms, and minimal changes to avoid confusion among the respondents of the questionnaires. The second stage involved the backward translation of the Arabic draft to English to check for any inconsistencies in the translation and assured each item's scientific contents individually. This stage was conducted by another three independent endocrinologists and an English linguistics expert.

Scale characteristics

The items' language format in both scales was put in either positive, negative, or mixed wording formats to elicit maximal responses. The maximal response scale constituted a 5-point Likert scale with easily understandable, comprehensible, and memorable options. The response time was proposed to be 15-30 minutes.

The Content Validity of the Items

We consulted 26 healthcare experts who were in direct contact with PCOS women in their daily practice to assess the content validity of the retrieved 67 items for married and 52 items for unmarried women separately. These experts included 11 gynecologists, six adult endocrinologists, six endocrine nurses, two dermatologists, and a psychiatrist. We adopted the content validity index (CVI) criteria of Lawshe (1975) [9], with its modification by Ayre and Scally (2014) [10]. Seventeen experts responded through different social media platforms, while the rest were directly interviewed.

A 4-point Likert scale was attached to each item to check whether the items were relevant to the concept, clearly understood, and simple to read. The CVI for each item was calculated according to the expert response, and only scores 3 and 4 were counted. We calculated the CVI for each item by dividing the number of experts who scored 3 or 4 for items by their total number. We accepted the items if the CVI was ≥0.65, which represented the lowest CVI as described by Ayre and Scally [10].

The next step was to test whether the items were essential to be asked in the context of PCOS, through the estimation of content validity ratio (CVR), which must be >0.37 for a total of 26 experts, as per Lawshe [9]. To achieve that, we attached a 3-point Likert scale wherein 1 represented "not necessary", 2 indicated "useful, but not necessary", and 3 stood for "essential or necessary". We counted only the scores of 3 in the CVR estimation.

The number of agreed-on items among the 26 experts, which achieved the required CVI and CVR, were 45 for unmarried women, and 57 for married women.

Distribution of the items in different domains

The resultant items were distributed according to their presumed relation to each other in the two five-domains questionnaires with a different number of items per domain for the unmarried and married women with PCOS as PCOSQoL-45 and PCOSQoL-57, respectively (Tables 1, 2). We tried to have uniform and homogenous domains with regard to the item count, which was achieved for PCOSQoL-57, but not for PCOSQoL-45 because the latter did not contain any queries about sexual life. The items across the domains had the same themes regardless of marital status, except for the items included in the sexual and fertility domain for the married women (Table 3).

**Table 1:** Initial draft of PCOSQoL-45 for unmarried women with PCOS PCOSQoL: Polycystic Ovary Syndrome Quality of Life

Domain	Code	Items
Psychological and emotional status (A)	A1	Suffered from bad mood due to PCOS?
	A2	Felt frequent tantrums due to PCOS?
	A3	Experienced trouble dealing with others?
	A4	Blamed yourself for having PCOS?
	A5	Suffered from low self-esteem due to PCOS?
	A6	Felt pessimistic about the treatment?
	A7	Felt easily tired?
	A8	Experienced fear of diseases such as diabetes, hypertension, and heart disease?
	A9	Felt the urge to abandon treatments because of repetitive visits to doctors?
Menstrual irregularities and fertility (B)	B1	Felt concerned about menstruation at long intervals?
	B2	Felt concerned about the complete cessation of menstruation?
	B3	Felt the regular need for oral contraceptive pills to control PCOS?
	B4	Felt concerned about future infertility?
	B5	Felt you would accept all other PCOS manifestations if assured of pregnancy?
	B6	Experienced feelings of fear of cancer due to PCOS?
	B7	Felt the need to decrease your weight to control PCOS?
Body image (C)	C1	Dissatisfied with some aspects of your appearance?
	C2	Spent a significant amount of time checking your appearance in the mirror?
	C3	Avoided looking at your appearance in the mirror?
	C4	Felt others are speaking negatively about your appearance?
	C5	Feared that others will discover flaws in your appearance?
	C6	Tried to hide some flaws in your appearance?
	C7	Ashamed of some part of your body?
	C8	Felt concerned about being overweight?
	C9	Felt concerned about a fast return to your previous weight after any weight loss?
Hair disorders and acne (D)	D1	Felt embarrassed about having excess facial and body hair?
	D2	Felt concerned about the progression pattern of excess body and facial hair?
	D3	Felt concerned about rapid re-growth of unwanted hair after its removal?
	D4	Felt the need to cover your body and face because of excess hair?
	D5	Felt that acne is affecting your appearance?
	D6	Felt that alopecia is affecting your appearance?
	D7	Felt that alopecia led to a decrease in your attraction and femininity?
	D8	Always wore a headscarf to cover your hair due to alopecia?
	D9	Felt that treatment of alopecia needs a long time and is worthless?
	D10	Avoided social circumstances due to alopecia?
	D11	Feared that facial acne will leave permanent scars?
Coping (E)	E1	Felt a lack of family support and acceptance of your disease?
	E2	Felt a lack of satisfaction with being a woman?
	E3	Concerned about your future role as a wife?
	E4	Felt difficulty in communicating with other women who have PCOS?
	E5	Felt disappointed about the cure?
	E6	Avoidance of social circumstances due to excess body hair?
	E7	Embarrassed to engage in social activities because of your appearance?
	E8	Compared your appearance with other women who you think are more physically attractive than you?
	E9	Tried to consult a medical expert about what you think is a flaw in your appearance?

**Table 2:** Initial draft of PCOSQoL-57 for married women with PCOS PCOSQoL: Polycystic Ovary Syndrome Quality of Life

Domain	Code	Items
Psychological and emotional status	A1	Suffered from bad mood due to PCOS?
	A2	Felt frequent tantrums due to PCOS?
	A3	Experienced trouble dealing with others?
	A4	Blamed yourself for having PCOS?
	A5	Suffered from low self-esteem due to PCOS?
	A6	Felt pessimistic about the treatment?
	A7	Felt easily tired?
	A8	Experienced fear of diseases such as diabetes, hypertension, and heart disease?
	A9	Felt the urge to abandon treatments because of repetitive visits to doctors?
	A10	Felt disappointed about the cure?
	A11	Felt a lack of family support and acceptance of your disease?
	A12	Felt difficulty in communicating with other women who have PCOS?
Fertility and sexual life	B1	Felt sad seeing children?
	B2	Felt sad seeing pregnant women?
	B3	Experienced concern about future infertility?
	B4	Felt fear of abortion?
	B5	Experienced fear of divorce or separation?
	B6	Felt useless relating to sexual intercourse due to infertility?
	B7	Felt unsatisfied with sexual life?
	B8	Felt a lack of sexual desire?
	B9	Felt dyspareunia during sexual intercourse?
	B10	Experienced a lack of orgasm?
	B11	Felt ashamed of sexual coldness/unresponsiveness?
Body image	C1	Dissatisfied with some aspects of your appearance?
	C2	Spent a significant amount of time checking your appearance in the mirror?
	C3	Avoided looking at your appearance in the mirror?
	C4	Felt others are speaking negatively about your appearance?
	C5	Feared that others will discover flaws in your appearance?
	C6	Tried to hide some flaws in your appearance?
	C7	Embarrassed to engage in social activities because of your appearance?
	C8	Ashamed of some part of your body?
	C9	Compared your appearance with other women who you think are more physically attractive than you?
	C10	Tried to consult a medical expert about what you think is a flaw in your appearance?
	C11	Experienced fear of treatment complications?
	C12	Felt a lack of satisfaction with being a woman?
Hair disorders and acne	D1	Felt embarrassed about having excess facial and body hair?
	D2	Felt concerned about the progression pattern of excess body and facial hair?
	D3	Felt concerned about rapid re-growth of unwanted hair after its removal?
	D4	Felt the need to cover your body and face because of excess hair?
	D5	Avoidance of social circumstances due to excess body hair?
	D6	Felt that acne is affecting your appearance?
	D7	Felt that alopecia is affecting your appearance?
	D8	Felt that alopecia led to a decrease in your attraction and femininity?
	D9	Always wore a headscarf or a veil to cover your hair due to alopecia?
	D10	Felt that treatment of alopecia needs a long time and is worthless?
	D11	Feared that facial acne will leave permanent scars?
	D12	Avoided social circumstances due to alopecia?
Obesity and menstrual disorders	E1	Felt concerned about being overweight?
	E2	Felt the need to decrease your weight to control PCOS?
	E3	Felt concerned about a fast return to your previous weight after any weight loss?
	E4	Concerned to reduce your weight to be more attractive for your husband?
	E5	Felt concerned about the complete cessation of menstruation?
	E6	Felt concerned about menstruation at long intervals?
	E7	Felt the regular need for oral contraceptive pills to control PCOS?
	E8	Experienced feelings of fear of cancer due to PCOS?
	E9	Felt you would accept all other PCOS manifestations if assured of pregnancy?
	E10	Felt a lack of satisfaction with your current role as a wife?

**Table 3:** The number of items per domain in the initial draft for both groups of women PCOSQoL: Polycystic Ovary Syndrome Quality of Life

Domains codes	PCOSQoL-45 domains	Items per domain (n=45)	PCOSQoL-57 domains	Items per domain (n=57)
A	Emotional and psychological	A1-A9	Emotional and psychological	A1-A12
B	Menstrual irregularities and fertility	B1-B7	Fertility and sexual life	B1-B11
C	Body image	C1-C9	Body image	C1-C12
D	Hair and acne	D1-D11	Hair and acne	D1-D12
E	Coping	E1-E9	Obesity and menstrual irregularities	E1-E10

Qualitative and quantitative assessment (April-July 2019)

To ensure that the items of the domain were appropriate, relevant, easily comprehensible, and understandable in terms of the focus and aim of the questionnaires for the women with PCOS, and to see whether these items genuinely reflected the presumed assessment for the HRQoL, we performed an initial qualitative, subjective assessment of the item pool through face validity. The purpose was to identify irrelevant items that could distract participants' attention and may result in omissions or faulty responses. Face validity provided a better chance for the future participants to complete the requested tasks, identified any wording ambiguities in any item, and pointed out any unconnected or missed concepts. Face validity is a qualitative and quantitative step to analyze questionnaire reliability [11,12].

To measure the impact score of the preliminary importance of the items as a measure for HRQoL, we attached a 5-point Likert scale for each item. We selected only those responses where the item was frequently perceived as important. The item impact score equals the frequency multiplied by the importance. We accepted all items with an impact score of ≥1.5.

Implementation of Face Validity Through Two Steps

The face validity was implemented through two steps. The first step involved 30 women with already diagnosed PCOS of different phenotypes from each group. We interviewed these women individually to obtain their opinion about the importance of the selected items. Each interview lasted about 15 minutes, and started with the question “What does PCOS mean to you?” or “Do you have any feeling regarding your diagnosis of PCOS?”, or “Did PCOS affect your quality of life by any means?”. The interviewer registered all those that looked important in the responses to be compared later with our preliminary list to see if it had any added value or new information. Then we gave each woman the specific numbered form and explained how to answer each item separately by encircling the score, which corresponds to its importance. After the completion, the authors asked for any further opinions about the items, to be written down or verbally registered.

The second step involved 30 healthcare professionals. They consisted of 10 adult endocrinologists, seven gynecologists, six endocrine nurses, three dermatologists, a psychiatrist, an internist, a family physician, and a gynecological radiologist. Twenty experts were approached through social media, and 10 experts responded in person. The same method described above was implemented for healthcare experts. 

We ensured the anonymity of the responses during the whole process. Each form was encoded as a number that was known only to the first authors.

In conclusion, we excluded four items from the form meant for the married women who did not achieve the required item impact score of ≥1.5. The four excluded items included two items from the emotional and psychological domain (A11 and A12), one item from the body image domain (C12), and one item from the hair and acne domain (D12). No item was excluded from the form for the unmarried women (Table 4).

**Table 4:** Impact factor for each item in the domains of PCOSQoL-45 and PCOSQoL-57 after face validation *These items had an impact factor <1.5, which will be deleted in subsequent analyses PCOSQoL: Polycystic Ovary Syndrome Quality of Life

PCOSQoL-45				PCOSQoL-57		
Domain	Code	Impact score		Domain	Code	Impact score
Psychological and emotional status	A1	2.28		Psychological and emotional status	A1	2.33
	A6	2.22			A7	2.33
	A9	2.08			A6	2.20
	A2	1.83			A10	2.20
	A7	2.17			A9	2.08
	A8	2.17			A2	2.05
	A3	1.87			A3	2.00
	A5	1.75			A4	2.00
	A4	1.70			A8	2.00
					A5	1.75
Menstrual irregularities and fertility	B7	2.67			A12*	1.45
	B4	2.63			A11*	1.42
	B2	2.42				
	B1	2.33		Fertility and sexual life	B4	2.83
	B3	2.28			B6	2.70
	B5	2.20			B2	2.67
	B6	2.08			B3	2.67
					B5	2.58
Body image	C1	2.80			B1	2.55
	C8	2.75			B8	2.55
	C6	2.50			B11	2.42
	C9	2.47			B7	2.42
	C2	2.33			B9	2.37
	C7	2.33			B10	2.22
	C5	2.22				
	C4	2.00		Body image	C10	2.97
	C3	1.83			C1	2.92
					C6	2.83
Hair disorders and acne	D1	2.97			C11	2.83
	D6	2.55			C8	2.70
	D2	2.42			C2	2.50
	D7	2.38			C7	2.45
	D3	2.22			C4	2.30
	D8	2.22			C9	2.23
	D11	2.12			C5	2.13
	D5	2.08			C3	1.75
	D9	2.00			C12*	1.48
	D4	1.97				
	D10	1.72		Hair disorders and acne	D3	2.95
					D2	2.88
Coping	E9	2.95			D6	2.72
	E7	2.38			D1	2.70
	E8	2.38			D8	2.58
	E5	2.12			D7	2.50
	E6	2.05			D4	2.42
	E1	1.87			D10	2.08
	E4	1.80			D11	2.03
	E2	1.78			D5	1.97
	E3	1.72			D9	1.80
					D12*	1.43
				Obesity and menstrual disorders	E1	3.20
					E2	3.13
					E3	2.92
					E5	2.92
					E6	2.78
					E4	2.67
					E7	2.67
					E9	2.23
					E8	2.13
					E10	1.83

The resultant final draft contained 45 items for unmarried women and 53 items for married women. This step helped us primarily to re-arrange the items in the domains sequentially according to their total mean score, presumed importance, and necessity, maintaining the same theme of the content. The new codes for the questionnaires at this stage were PCOSQoL-45 for unmarried, and PCOSQoL-53 for married women with PCOS.

The second draft (Tables 5, 6)

The questionnaires were rearranged in a clear paper-pack format with five domains for each group of women with PCOS. Each item was attached with a 5-point Likert scale, where 1 denoted that the complaint item was "always" present, and (5) denoted that this complaint item was "never" perceived. The item scores in between correspond to the frequency of the women's complaint item in the last two weeks. The response was requested for each item accordingly. The respondent had the right to omit any item that they found irrelevant or embarrassing. On completion of the questionnaire, the overall score and the score for each domain were marked. The interviewer did the marking with anonymous record registration.

The questionnaires would be assessed through a psychometric validation process and statistical tests of reliability, including test-retest, internal consistency, and construct validity, to assess the questionnaire’s acceptability, responsiveness, and interpretability in a specific population [12]. And to achieve that, we performed a real-world study of reliability testing.

**Table 5:** Second draft for unmarried women with PCOS (PCOSQoL-45) PCOSQoL: Polycystic Ovary Syndrome Quality of Life

Domain	Code	Items	Never	Seldom	Quite often	Very often	Always
Psychological and emotional status	A1	Suffered from bad mood due to PCOS?	5	4	3	2	1
	A6	Felt pessimistic about the treatment?	5	4	3	2	1
	A9	Felt the urge to abandon treatments because of repetitive visits to doctors?	5	4	3	2	1
	A2	Felt frequent tantrums due to PCOS?	5	4	3	2	1
	A7	Felt easily tired?	5	4	3	2	1
	A8	Experienced fear of diseases such as diabetes, hypertension, and heart disease?	5	4	3	2	1
	A3	Experienced trouble dealing with others?	5	4	3	2	1
	A5	Suffered from low self-esteem due to PCOS?	5	4	3	2	1
	A4	Blamed yourself for having PCOS?	5	4	3	2	1
Menstrual irregularities and fertility	B7	Felt the need to decrease your weight to control PCOS?	5	4	3	2	1
	B4	Felt concerned about future infertility?	5	4	3	2	1
	B2	Felt concerned about the complete cessation of menstruation?	5	4	3	2	1
	B1	Felt concerned about menstruation at long intervals?	5	4	3	2	1
	B3	Felt the regular need for oral contraceptive pills to control PCOS?	5	4	3	2	1
	B5	Felt you would accept all other PCOS manifestations if assured of pregnancy?	5	4	3	2	1
	B6	Experienced feelings of fear of cancer due to PCOS?	5	4	3	2	1
Body image	C1	Dissatisfied with some aspects of your appearance?	5	4	3	2	1
	C8	Felt concerned about being overweight?	5	4	3	2	1
	C6	Tried to hide some flaws in your appearance?	5	4	3	2	1
	C9	Felt concerned about a fast return to your previous weight after any weight loss?	5	4	3	2	1
	C2	Spent a significant amount of time checking your appearance in the mirror?	5	4	3	2	1
	C7	Ashamed of some part of your body?	5	4	3	2	1
	C5	Feared that others will discover flaws in your appearance?	5	4	3	2	1
	C4	Felt others are speaking negatively about your appearance?	5	4	3	2	1
	C3	Avoided looking at your appearance in the mirror?	5	4	3	2	1
Hair disorders and acne	D1	Felt embarrassed about having excess facial and body hair?	5	4	3	2	1
	D6	Felt that alopecia is affecting your appearance?	5	4	3	2	1
	D2	Felt concerned about the progression pattern of excess body and facial hair?	5	4	3	2	1
	D7	Felt that alopecia led to a decrease in your attraction and femininity?	5	4	3	2	1
	D3	Felt concerned about rapid re-growth of unwanted hair after its removal?	5	4	3	2	1
	D8	Always wore a headscarf to cover your hair due to alopecia?	5	4	3	2	1
	D11	Fear from facial acne to leave permanent scars?	5	4	3	2	1
	D5	Felt that acne is affecting your appearance?	5	4	3	2	1
	D9	Felt that treatment of alopecia needs a long time and is worthless?	5	4	3	2	1
	D4	Felt the need to cover your body and face because of excess hair?	5	4	3	2	1
	D10	Avoided social circumstances due to alopecia?	5	4	3	2	1
Coping	E9	Tried to consult a medical expert about what you think is a flaw in your appearance?	5	4	3	2	1
	E7	Embarrassed to engage in social activities because of your appearance?	5	4	3	2	1
	E8	Compared your appearance with other women who you think are more physically attractive than you?	5	4	3	2	1
	E5	Felt disappointed about the cure?	5	4	3	2	1
	E6	Avoidance of social circumstances due to excess body hair?	5	4	3	2	1
	E1	Felt a lack of family support and acceptance of your disease?	5	4	3	2	1
	E4	Felt difficulty in communicating with other women who have PCOS?	5	4	3	2	1
	E2	Felt a lack of satisfaction with being a woman?	5	4	3	2	1
	E3	Concerned about your future role as a wife?	5	4	3	2	1

**Table 6:** Second draft for married women with PCOS (PCOSQoL-53) PCOSQoL: Polycystic Ovary Syndrome Quality of Life

Domain	Code	Items	Never	Seldom	Quite often	Very often	Always
Psychological and emotional status	A1	Suffered from bad mood due to PCOS?	5	4	3	2	1
	A7	Felt easily tired?	5	4	3	2	1
	A6	Felt pessimistic about the treatment?	5	4	3	2	1
	A10	Felt disappointed about the cure?	5	4	3	2	1
	A9	Felt the urge to abandon treatments because of repetitive visits to doctors?	5	4	3	2	1
	A2	Felt frequent tantrums due to PCOS?	5	4	3	2	1
	A3	Experienced trouble dealing with others?	5	4	3	2	1
	A4	Blamed yourself for having PCOS?	5	4	3	2	1
	A8	Experienced fear of diseases such as diabetes, hypertension, and heart disease?	5	4	3	2	1
	A5	Suffered from low self-esteem due to PCOS?	5	4	3	2	1
Fertility and sexual life	B4	Felt fear of abortion?	5	4	3	2	1
	B6	Felt useless regarding sexual intercourse due to infertility?	5	4	3	2	1
	B2	Felt sad seeing pregnant women?	5	4	3	2	1
	B3	Experienced concern about future infertility?	5	4	3	2	1
	B5	Experienced fear of divorce or separation?	5	4	3	2	1
	B1	Felt sad seeing children?	5	4	3	2	1
	B8	Felt a lack of sexual desire?	5	4	3	2	1
	B11	Felt ashamed of sexual coldness/unresponsiveness?	5	4	3	2	1
	B7	Felt unsatisfied with sexual life?	5	4	3	2	1
	B9	Felt dyspareunia during sexual intercourse?	5	4	3	2	1
	B10	Experienced a lack of orgasm?	5	4	3	2	1
Body image	C10	Try to consult a medical expert about what you think is a flaw in your appearance?	5	4	3	2	1
	C1	Dissatisfied with some aspects of your appearance?	5	4	3	2	1
	C6	Tried to hide some flaws in your appearance?	5	4	3	2	1
	C11	Experienced fear of treatment complications?	5	4	3	2	1
	C8	Ashamed of some part of your body?	5	4	3	2	1
	C2	Spent a significant amount of time checking your appearance in the mirror?	5	4	3	2	1
	C7	Embarrassed to engage in social activities because of your appearance?	5	4	3	2	1
	C4	Felt others are speaking negatively about your appearance?	5	4	3	2	1
	C9	Compared your appearance with other women who you think are more physically attractive than you?	5	4	3	2	1
	C5	Feared that others will discover flaws in your appearance?	5	4	3	2	1
	C3	Avoided looking at your appearance in the mirror?	5	4	3	2	1
Hair disorders and acne	D3	Felt concerned about rapid re-growth of unwanted hair after its removal?	5	4	3	2	1
	D2	Felt concerned about the progression pattern of excess body and facial hair?	5	4	3	2	1
	D6	Felt that acne is affecting your appearance?	5	4	3	2	1
	D1	Felt embarrassed about having excess facial and body hair?	5	4	3	2	1
	D8	Felt that alopecia led to a decrease in your attraction and femininity?	5	4	3	2	1
	D7	Felt that alopecia is affecting your appearance?	5	4	3	2	1
	D4	Felt the need to cover your body and face because of excess hair?	5	4	3	2	1
	D10	Felt that treatment of alopecia needs a long time and is worthless?	5	4	3	2	1
	D11	Feared that facial acne will leave permanent scars?	5	4	3	2	1
	D5	Avoidance of social circumstances due to excess body hair?	5	4	3	2	1
	D9	Always wore a headscarf or a veil to cover your hair due to alopecia?	5	4	3	2	1
Obesity and menstrual disorders	E1	Felt concerned about being overweight?	5	4	3	2	1
	E2	Felt the need to decrease your weight to control PCOS?	5	4	3	2	1
	E3	Felt concerned about a fast return to your previous weight after any weight loss?	5	4	3	2	1
	E5	Felt concerned about the complete cessation of menstruation?	5	4	3	2	1
	E6	Felt concerned about menstruation at long intervals?	5	4	3	2	1
	E4	Concerned to reduce your weight to be more attractive for your husband?	5	4	3	2	1
	E7	Felt the regular need for oral contraceptive pills to control PCOS?	5	4	3	2	1
	E9	Felt you would accept all other PCOS manifestations if assured of pregnancy?	5	4	3	2	1
	E8	Experienced feelings of fear of cancer due to PCOS?	5	4	3	2	1
	E10	Felt a lack of satisfaction with your current role as a wife?	5	4	3	2	1

Initial recruitment in a cross-sectional preliminary pilot study

The inclusion criteria were simple and included all premenopausal women with the diagnosis of PCOS according to the Rotterdam criteria (2003) [1], within the age range of 16-40 years who attended Faiha Specialized Diabetes Endocrine and Metabolism Center (FDEMC) in Basrah, Southern Iraq, for diagnosis and follow-up, and who were able to comprehend the questionnaire individually or with (independent) assistance. Patients with any comorbidity not related to PCOS and pregnant women were excluded.

One of the research team members approached individual women to explain the questionnaire items to help solve any difficulty in understanding them. Participants were made aware that this questionnaire would not be a part of their ongoing care, and declining to participate would not compromise their care level. The participants were asked to sign an informed consent form that was provided and explained by the interviewer before the enrollment. The participants were also told that they could omit the answer for any question they found bothersome or embarrassing. They could exclude themselves from the study at any point in the future according to their will.

Baseline clinical and demographic data were collected from electronic clinical records, with the assigned case numbers for de-identification and confidentiality. The participants were expected to answer the items directly by encircling the most appropriate answer for what they felt during the last two weeks. The exact timing of the response would be calculated. The proposed time was around 15-30 minutes.

This pilot study, which lasted for five days, helped us identify any logistical difficulties in implementing the questionnaire by both the women and the research team. To achieve this goal, we initially enrolled 30 women with PCOS from each group, who agreed to answer the questionnaire according to the previously mentioned instructions. After the conclusion of the pilot study, no change had been made to the structures of the questionnaires. The next step was to measure questionnaires' reliability on a larger scale.

Phase 2: Statistical analysis of reliability

Reliability measures the questionnaire's reproducibility for different individuals or in various situations by measuring the presumed outcome, which should be the same irrespective of the change in respondents [13].

Test-retest reliability

Test-retest reliability is useful to measure the stability of the HRQoL questionnaires on different occasions [13].

The retest evaluation was scheduled within five to seven days from the initial interview (test). We enrolled women with PCOS who attended FDEMC within the next 60 days. The timing between the test and retest was planned to minimize any recall bias, i.e., patients memorizing their initial responses without reading the questionnaire items on retest occasion.

Participants would not receive any treatment intervention that would alter their HRQoL during these five to seven days. The duration between the first and second visits was determined by the women receiving the results of their investigation by the central endocrine laboratory. We enrolled 195 married women and 173 unmarried women with PCOS, who consented to the test-retest reliability evaluation, to answer the PCOSQoL-53 and PCOSQoL-45, respectively.

The Spearman's correlational analysis rho (ρ) and Wilcoxon nonparametric signed-rank test were used to calculating the statistical differences between the two occasions' ordinal scores. We calculated the two-way mixed intraclass correlations (ICC) coefficients to evaluate for any significant relationship (p: ≤0.05) between the scores at the two times and draw a heat map for the items according to their corresponding score. The coefficient values of >0.7 were considered good, >0.8 were optimal, and >0.9 were determined excellent to measure the ICC [14].

Internal consistency

The statistical method used to measure the association of the items within the scale was Cronbach's alpha reliability coefficient, which ranges between zero and one. Scores of >0.7 meant that the questionnaire items were measuring related constructs. Scores that were very close to 1 indicated higher redundancy of the scale of the item. We recalculated the Cronbach's alpha after removing individual domain items with low scores, to make the scale more reliable. If the alpha coefficient score increased after a question was removed, this would mean that the item had no correlation with other items in the domain. This process measured the item-total consistency and internal reliability of the items (Presentation: Gliem JA, Gliem RR. Calculating, interpreting, and reporting Cronbach’s alpha reliability coefficient for Likert-type scales. 2003 Midwest Research to Practice Conference in Adult, Continuing and Community Education. Columbus, OH). We also calculated the inter-item correlations for each domain's items, intending to reach a corrected inter-item correlation mean of more than 0.3. The inter-rater reliability analysis was also calculated.

Upon completing these two steps of the test-retest study, which could also be considered an item reduction step, we excluded six items from the PCOSQoL-53 (A10, B9, E1, E2, E4, and E6) to reduce the items to 47, and the questionnaire was renamed as (PCOSQoL-47). We excluded three items from the PCOSQoL-45 (A7, C8, and C9) to bring the items down to 42, and the questionnaire was renamed a (PCOSQoL-42). The third draft of both questionnaires was created (Tables 7, 8).

**Table 7:** Third draft for unmarried women with PCOS (PCOSQoL-42) after the end of test-retest reliability evaluation PCOSQoL: Polycystic Ovary Syndrome Quality of Life

Domain	Code	Items	Never	Seldom	Quite often	Very often	Always
Psychological and emotional status	A1	Suffered from bad mood due to PCOS?	5	4	3	2	1
	A6	Felt pessimistic about the treatment?	5	4	3	2	1
	A9	Felt the urge to abandon treatments because of repetitive visits to doctors?	5	4	3	2	1
	A2	Felt frequent tantrums due to PCOS?	5	4	3	2	1
	A8	Experienced fear of diseases such as diabetes, hypertension, and heart disease?	5	4	3	2	1
	A3	Experienced trouble dealing with others?	5	4	3	2	1
	A5	Suffered from low self-esteem due to PCOS?	5	4	3	2	1
	A4	Blamed yourself for having PCOS?	5	4	3	2	1
Menstrual irregularities and fertility	B7	Felt the need to decrease your weight to control PCOS?	5	4	3	2	1
	B4	Felt concerned about future infertility?	5	4	3	2	1
	B2	Felt concerned about the complete cessation of menstruation?	5	4	3	2	1
	B1	Felt concerned about menstruation at long intervals?	5	4	3	2	1
	B3	Felt the regular need for oral contraceptive pills to control PCOS?	5	4	3	2	1
	B5	Felt you would accept all other PCOS manifestations if assured of pregnancy?	5	4	3	2	1
	B6	Experienced feelings of fear of cancer due to PCOS?	5	4	3	2	1
Body image	C1	Dissatisfied with some aspects of your appearance?	5	4	3	2	1
	C6	Tried to hide some flaws in your appearance?	5	4	3	2	1
	C2	Spent a significant amount of time checking your appearance in the mirror?	5	4	3	2	1
	C7	Ashamed of some part of your body?	5	4	3	2	1
	C5	Feared that others will discover flaws in your appearance?	5	4	3	2	1
	C4	Felt that others are speaking negatively about your appearance?	5	4	3	2	1
	C3	Avoided looking at your appearance in the mirror?	5	4	3	2	1
Hair disorders and acne	D1	Felt embarrassed about having excess facial and body hair?	5	4	3	2	1
	D6	Felt that alopecia is affecting your appearance?	5	4	3	2	1
	D2	Felt concerned about the progression pattern of excess body and facial hair?	5	4	3	2	1
	D7	Felt that alopecia led to a decrease in your attraction and femininity?	5	4	3	2	1
	D3	Felt concerned about rapid re-growth of unwanted hair after its removal?	5	4	3	2	1
	D8	Always wore a headscarf to cover your hair due to alopecia?	5	4	3	2	1
	D11	Fear from facial acne to leave permanent scars?	5	4	3	2	1
	D5	Felt that acne is affecting your appearance?	5	4	3	2	1
	D9	Felt that treatment of alopecia needs a long time and is worthless?	5	4	3	2	1
	D4	Felt the need to cover your body and face because of excess hair?	5	4	3	2	1
	D10	Avoid the social circumstances due to alopecia?	5	4	3	2	1
Coping	E9	Tried to consult a medical expert about what you think is a flaw in your appearance?	5	4	3	2	1
	E7	Embarrassed to engage in social activities because of your appearance?	5	4	3	2	1
	E8	Compared your appearance with other women who you think are more physically attractive than you?	5	4	3	2	1
	E5	Felt disappointed about the cure?	5	4	3	2	1
	E6	Avoidance of social circumstances due to excess body hair?	5	4	3	2	1
	E1	Felt a lack of family support and acceptance of your disease?	5	4	3	2	1
	E4	Felt difficulty in communicating with other women who have PCOS?	5	4	3	2	1
	E2	Felt a lack of satisfaction with being a woman?	5	4	3	2	1
	E3	Concerned about your future role as a wife?	5	4	3	2	1

**Table 8:** Third draft for married women with PCOS (PCOSQoL-47) after the end of test-retest reliability evaluation PCOSQoL: Polycystic Ovary Syndrome Quality of Life

Domain	Item code	Items	Never	Seldom	Quite often	Very often	Always
Psychological and emotional status	A1	Suffered from bad mood due to PCOS?	5	4	3	2	1
	A7	Felt easily tired?	5	4	3	2	1
	A6	Felt pessimistic about the treatment?	5	4	3	2	1
	A9	Felt the urge to abandon treatments because of repetitive visits to doctors?	5	4	3	2	1
	A2	Felt frequent tantrums due to PCOS?	5	4	3	2	1
	A3	Experienced trouble dealing with others?	5	4	3	2	1
	A4	Blamed yourself for having PCOS?	5	4	3	2	1
	A8	Experienced fear of diseases such as diabetes, hypertension, and heart disease?	5	4	3	2	1
	A5	Suffered from low self-esteem due to PCOS?	5	4	3	2	1
Fertility and sexual life	B4	Felt fear of abortion?	5	4	3	2	1
	B6	Felt useless regarding sexual intercourse due to infertility?	5	4	3	2	1
	B2	Felt sad seeing pregnant women?	5	4	3	2	1
	B3	Experienced concern about future infertility?	5	4	3	2	1
	B5	Experienced fear of divorce or separation?	5	4	3	2	1
	B1	Felt sad seeing children?	5	4	3	2	1
	B8	Felt a lack of sexual desire?	5	4	3	2	1
	B11	Felt ashamed of sexual coldness/unresponsiveness?	5	4	3	2	1
	B7	Felt unsatisfied with sexual life?	5	4	3	2	1
	B10	Experienced a lack of orgasm?	5	4	3	2	1
Body image	C10	Tried to consult a medical expert about what you think is a flaw in your appearance?	5	4	3	2	1
	C1	Dissatisfied with some aspects of your appearance?	5	4	3	2	1
	C6	Tried to hide some flaws in your appearance?	5	4	3	2	1
	C11	Experienced fear of treatment complications?	5	4	3	2	1
	C8	Ashamed of some part of your body?	5	4	3	2	1
	C2	Spent a significant amount of time checking your appearance in the mirror?	5	4	3	2	1
	C7	Embarrassed to engage in social activities because of your appearance?	5	4	3	2	1
	C4	Felt others are speaking negatively about your appearance?	5	4	3	2	1
	C9	Compared your appearance with other women who you think are more physically attractive than you?	5	4	3	2	1
	C5	Feared that others will discover flaws in your appearance?	5	4	3	2	1
	C3	Avoided looking at your appearance in the mirror?	5	4	3	2	1
Hair disorders and acne	D3	Felt concerned about rapid re-growth of unwanted hair after its removal?	5	4	3	2	1
	D2	Felt concerned about the progression pattern of excess body and facial hair?	5	4	3	2	1
	D6	Felt that acne is affecting your appearance?	5	4	3	2	1
	D1	Felt embarrassed about having excess facial and body hair?	5	4	3	2	1
	D8	Felt that alopecia led to a decrease in your attraction and femininity?	5	4	3	2	1
	D7	Felt that alopecia is affecting your appearance?	5	4	3	2	1
	D4	Felt the need to cover your body and face because of excess hair?	5	4	3	2	1
	D10	Felt that treatment of alopecia needs a long time and is worthless?	5	4	3	2	1
	D11	Feared that facial acne will leave permanent scars?	5	4	3	2	1
	D5	Avoidance of social circumstances due to excess body hair?	5	4	3	2	1
	D9	Always wore a headscarf or a veil to cover your hair due to alopecia?	5	4	3	2	1
Obesity and menstrual disorders	E3	Felt concerned about a fast return to your previous weight after any weight loss?	5	4	3	2	1
	E5	Felt concerned about the complete cessation of menstruation?	5	4	3	2	1
	E7	Felt the regular need for oral contraceptive pills to control PCOS?	5	4	3	2	1
	E9	Felt you would accept all other PCOS manifestations if assured of pregnancy?	5	4	3	2	1
	E8	Experienced feelings of fear of cancer due to PCOS?	5	4	3	2	1
	E10	Felt a lack of satisfaction with your current role as a wife?	5	4	3	2	1

Gathering of the data sets

For every respondent, there were scores of either PCOSQoL-47 or PCOSQoL-42 and the WHOQOL-BREF. The questionnaires' data were captured directly on an already prepared IBM SPSS Statistics for Windows, Version 26.0. (IBM Corp., Armonk, NY) format by the interviewer. It was then checked and compared by two other research members for consistency and accuracy of the data.

The statistical analysis was done using the same version of SPSS; we performed the nonparametric correlation, bivariate correlation, and reliability construct validity analysis at a two-tailed significance level of ≤0.05.

All the paper forms were sorted and stored according to the first author's registration numbers, and they were kept ready to be retrieved on request from any respondent. All enrolled women were provided with a copy of their responses to ensure transparency in dealing with their data. All women were told that they would be free to withhold their consent at any time during the study till the time of final publication, and this would not affect in any way the level of medical care provided for them in FDEMC. However, none of the women withheld their consent.

Ethical approval

All the study phases which involved interviewing women with PCOS were in accordance with the ethical standards of the Faiha Specialized Diabetes Endocrine and Metabolism Center Research Committee, from which the ethical approval was obtained, and with the 1964 Declaration of Helsinki and its later amendments or comparable ethical standards.

Informed consent

Informed consent was obtained from all individual participants included in the study, during all phases of the study.

## Results

Phase 1

After the initial steps of item retrieval, the items-reduction phases were pivotal in determining the construct dimensions, questionnaire's items development and format, and questionnaire length. The first draft of the items for the questionnaires in Arabic was created after an exhaustive analysis of content validity. The first draft for unmarried women contained 45 items and was encoded as PCOSQoL-45, while that of married women contained 57 items and was encoded as PCOSQoL-57 (Tables 1-4). The mean CVI and CVR for the PCOSQoL-45 were 0.94 and 0.92, and those for PCOSQoL-57 were 0.92 and 0.93, respectively.

The face validity analysis for both questionnaires, which was done through 30 women with PCOS from each group along with 30 healthcare experts, created the second drafts for the questionnaires. No change was carried out for the PCOSQoL-45, unlike PCOSQoL-57, from which four items were omitted, to be renamed as PCOSQoL-53 (Tables 5, 6).

The pilot study

The resultant draft was used in the five-day pilot study on 30 women with PCOS from each group and was attached to a five-point Likert scale. The score of 1 denoted that the complaint item was always present, and 5 denoted that this complaint item was never perceived in the last two weeks before the presentation (Tables 5, 6). The pilot study pointed out some logistical difficulties in implementing the questionnaires for women with PCOS. It gave us an idea about the general characteristics of women, which would be considered in the next steps. The general characteristics of women in the pilot study are summarized in Table 9.

**Table 9:** General characteristics of the women with PCOS in the pilot study BMI: body mass index; PCOSQoL: Polycystic Ovary Syndrome Quality of Life; SD: standard deviation; SE: standard error

Variables		PCOSQoL-45	PCOSQoL-53
Mean age (years) ± SD		22.53 ± 4.10	24.50 ± 5.09
Mean BMI (kg/m^2^) ± SD		27.63 ± 3.50	29.09 ±4.28
Median duration of PCOS ± SE		3.40 ± 1.92	3.94 ± 2.13
Number of women with total item response (%)		27 (90.00)	28 (93.33)
Mean response time (minutes)	Mean ± SD	20 ± 4	22 ± 4
	Maximum	29	30
	Minimum	15	15

Phase 2: test-retest reliability

After the conclusion of the pilot study, we performed test-retest reliability analysis to assess for the questionnaires' stability over time in measuring the given outcome, where we used the same draft of the questionnaire twice for the same woman, to be responded to in the enrollment visit, and after five to seven days.

We enrolled 173 unmarried and 195 married women to respond to PCOSQoL-45 and PCOSQoL-53, respectively. No woman had been excluded or none had defaulted during the test-retest evaluation. Table 10 presents the general characteristics of enrolled women in the test-retest reliability evaluation. The response rate of women with each item is presented in Table 11.

**Table 10:** General characteristics of the women with PCOS in the test re-test reliability evaluation using PCOSQoL-45 and PCOSQoL-53 BMI: body mass index; PCOSQoL: Polycystic Ovary Syndrome Quality of Life; SD: standard deviation; SE: standard error

Variables		PCOSQoL-45 (n=173)			PCOSQoL-53 (n=195)	
		Test	Retest		Test	Retest
Mean age (years) ± SD		23.45 ± 4.65			29.30 ± 5.87	
Mean BMI (kg/m^2^) ± SD		28.45 ± 5.82			31.57 ± 5.86	
Median duration of PCOS ± SE		3.00 ± .27			12.00 ± .44	
Mean response time (minutes)	Mean ± SD	20 ± 4	21 ± 4		21 ± 4	22 ± 4
	Range	15–29	15–30		15–30	16–30
Response rate per domain, n (%)	Domain A	164 (94.80)	116 (67.05)		172 (88.21)	180 (92.30)
	Domain B	167 (96.53)	137 (79.19)		122 (62.54)	133 (68.21)
	Domain C	170 (98.27)	114 (65.90)		163 (83.59)	175 (89.74)
	Domain D	161 (93.06)	171 (98.84)		162 (83.08)	179 (91.80)
	Domain E	156 (90.17)	156 (90.17)		139 (71.28)	144 (73.85)
	Total	143 (82.66)	67 (38.73)		95 (48.72)	104 (53.33)
	All test and retest	61 (35.26)			95 (48.72)	

**Table 11:** Item by item response and Spearman's correlation coefficient (ρ) of the women with PCOS in the test re-test reliability evaluation using PCOSQoL-45 and PCOSQoL-53 The two-tailed significance is >0.001 in all items and all domains PCOSQoL: Polycystic Ovary Syndrome Quality of Life

PCOSQoL-45 (n=173)						PCOSQoL-53 (n=195)				
Domain	Item code	Test, n (%)	Retest, n (%)	Spearman's correlation coefficient (ρ)		Domain	Item code	Test, n (%)	Retest, n (%)	Spearman's correlation coefficient (ρ)
Psychological and emotional status	A1	173 (100)	173 (100)	.89		Psychological and emotional status	A1	188 (96.41)	195 (100)	.92
	A2	173 (100)	167 (96.53)	.92			A2	187 (95.90)	194 (99.49)	.86
	A3	169 (97.69)	165 (95.38)	.85			A3	192 (98.46)	194 (99.49)	.87
	A4	173 (100)	165 (95.38)	.91			A4	177 (90.77)	195 (100)	.99
	A5	170 (98.27)	160 (92.49)	.86			A5	175 (89.74)	195 (100)	.90
	A6	172 (99.42)	161 (93.06)	.89			A6	178 (91.28)	194 (99.49)	.90
	A7	173 (100)	165 (95.38)	.85			A7	184 (94.36)	195 (100)	.89
	A8	173 (100)	167 (96.53)	.91			A8	186 (95.39)	194 (99.49)	.91
	A9	169 (97.69)	161 (93.06)	.93			A9	184 (94.36)	189 (96.92)	.85
	Total	164 (94.80)	116 (67.05)	.96			A10	180 (92.30)	186 (95.39)	.90
							Total	172 (88.21)	180 (92.30)	.96
Menstrual irregularities and fertility	B1	173 (100)	168 (97.11)	.92						
	B2	173 (100)	169 (97.69)	.90		Fertility and sexual life	B1	171 (87.69)	179 (91.80)	.91
	B3	171 (98.84)	167 (96.53)	.97			B2	168 (86.15)	178 (91.28)	.90
	B4	172 (99.42)	169 (97.69)	.88			B3	173 (88.72)	186 (95.39)	.87
	B5	167 (96.53)	162 (93.64)	.96			B4	166 (85.13)	172 (88.21)	.92
	B6	172 (99.42)	167 (96.53)	.90			B5	173 (88.72)	179 (91.80)	.92
	B7	173 (100)	165 (95.38)	.93			B6	165 (84.62)	177 (90.77)	.91
	Total	167 (96.53)	137 (79.19)	.97			B7	173 (88.72)	182 (93.33)	.89
							B8	182 (93.33)	189 (96.92)	.92
Body image	C1	173 (100)	163 (94.22)	.84			B9	181 (92.82)	190 (97.44)	.83
	C2	173 (100)	161 (93.06)	.86			B10	180 (92.30)	189 (96.92)	.89
	C3	173 (100)	166 (95.95)	.93			B11	183 (93.85)	189 (96.92)	.92
	C4	173 (100)	166 (95.95)	.87			Total	122 (62.54)	133 (68.21)	.96
	C5	171 (98.84)	162 (93.64)	.84						
	C6	173 (100)	162 (93.64)	.88		Body image	C1	190 (97.44)	193 (98.97)	.87
	C7	173 (100)	168 (97.11)	.94			C2	189 (96.92)	195 (100)	.82
	C8	173 (100)	168 (97.11)	.91			C3	190 (97.44)	193 (98.97)	.94
	C9	172 (99.42)	171 (98.84)	.81			C4	187 (95.90)	195 (100)	.89
	Total	170 (98.27)	114 (65.90)	.94			C5	190 (97.44)	195 (100)	.90
							C6	193 (98.97)	193 (98.97)	.95
Hair disorders and acne	D1	173 (100)	173 (100)	.82			C7	192 (98.46)	194 (99.49)	.92
	D2	173 (100)	172 (99.42)	.81			C8	191 (98.00)	194 (99.49)	.91
	D3	173 (100)	173 (100)	.87			C9	191 (98.00)	192 (98.46)	.88
	D4	172 (99.42)	173 (100)	.88			C10	188 (96.41)	191 (98.00)	.89
	D5	173 (100)	172 (99.42)	.84			C11	168 (86.15)	179 (91.80)	.88
	D6	173 (100)	173 (100)	.80			Total	163 (83.59)	175 (89.74)	.93
	D7	171 (98.84)	173 (100)	.86						
	D8	172 (99.42)	173 (100)	.90		Hair disorders and acne	D1	185 (94.87)	185 (94.87)	.93
	D9	171 (98.84)	173 (100)	.94			D2	194 (99.49)	195 (100)	.90
	D10	167 (96.53)	173 (100)	.91			D3	193 (98.97)	195 (100)	.89
	D11	171 (98.84)	173 (100)	.80			D4	193 (98.97)	195 (100)	.90
	Total	161 (93.06)	171 (98.84)	.94			D5	193 (98.97)	195 (100)	.83
							D6	185 (94.87)	195 (100)	.95
Coping	E1	164 (94.80)	172 (99.42)	.86			D7	189 (96.92)	195 (100)	.86
	E2	169 (97.69)	173 (100)	.83			D8	185 (94.87)	195 (100)	.96
	E3	170 (98.27)	173 (100)	.86			D9	193 (98.97)	195 (100)	.85
	E4	167 (96.53)	170 (98.27)	.93			D10	183 (93.85)	194 (99.49)	.88
	E5	170 (98.27)	173 (100)	.96			D11	178 (91.28)	190 (97.44)	.88
	E6	173 (100)	173 (100)	.96			Total	162 (83.08)	179 (91.80)	.95
	E7	173 (100)	171 (98.84)	.93						
	E8	173 (100)	170 (98.27)	.91		Obesity and menstrual disorders	E1	191 (98.00)	193 (98.97)	.86
	E9	171 (98.84)	165 (95.38)	.84			E2	181 (92.82)	181 (92.82)	.87
	Total	156 (90.17)	156 (90.17)	.96			E3	186 (95.39)	186 (95.39)	.92
Overall total		143 (82.66)	67 (38.73)	.98			E4	182 (93.33)	188 (96.41)	.91
							E5	184 (94.36)	192 (98.46)	.92
							E6	189 (96.92)	191 (98.00)	.91
							E7	173 (88.72)	182 (93.33)	.90
							E8	180 (92.30)	195 (100)	.91
							E9	161 (82.56)	164 (84.10)	.93
							E10	182 (93.33)	182 (93.33)	.90
							Total	139 (71.28)	144 (73.85)	.90
						Overall total		95 (48.72)	104 (53.33)	.96

Among respondents to the PCOSQoL-45 test phase, the overall response rate was around 83%, ranging from 90% for the coping domain to 98% for the body image domain. The maximal drop-out in the test phase was for item E1 (Felt a lack of family support and acceptance of your disease?), where nine women declined to respond, with the least response rate of 95%. The overall response rate was around 39% for the retesting phase, with a range from 66% for the body image domain to 99% for the hair disorders and acne domain. The maximal drop-out in the retest phase was for item A5 (Suffered from low self-esteem due to PCOS?), where 13 women declined to respond, with the least response rate of 92.5%. Overall, only 61 women responded to all items during the test and retest phases, representing only 35%.

**Table 12:** Wilcoxon nonparametric signed-rank test and Spearman's correlation analysis for the test-retest reliability analysis *The two-tailed significance level was less than 0.001 PCOSQoL: Polycystic Ovary Syndrome Quality of Life; SD: standard deviation

Domain	PCOSQoL-45				PCOSQoL-53		
	Test mean score ± SD	Retest mean score ± SD	Spearman's correlation coefficient (ρ)*		Test mean score ± SD	Retest mean score ± SD	Spearman's correlation coefficient (ρ)*
A	2.15 ± .82	2.26 ± .72	.96		2.34 ± .83	2.49 ± .77	.96
B	2.28 ± .87	2.32 ± .89	.97		2.28 ± 1.04	2.37 ± .89	.96
C	2.27 ± .84	2.41 ± .82	.94		2.35 ± .90	2.50 ± .81	.93
D	2.02 ± .63	2.09 ± .61	.94		2.14 ± .75	2.18 ± .67	.95
E	2.37 ± 1.07	2.51 ± .97	.96		2.01 ± .42	2.05 ± .39	.90
Total	2.18 ± .71	2.32 ± .64	.98		2.11 ± .52	2.22 ± .49	.96

For respondents to PCOSQoL-53, the overall response rate was around 49% for the test vs. 53% in the retest phase, with a range from 63% to 88% for the fertility and sexual life domain and for the psychological and emotional status domain, respectively, which was similar to the response rate in retest (68% to 92% for the fertility and sexual life domain and psychological and emotional status domain), respectively. The maximal drop-out item in the test and retest phases was item E9 (Felt you would accept all other PCOS manifestations if assured of pregnancy?) where 34 women declined to respond in the test phase, with the least response rate of 83%, vs. 31 women in retest phase with the least response rate of 84%. Overall, only 95 women responded to all items during the test and retest phases, representing only 49%.

For both PCOSQol-45 and PCOSQoL-53, using the Wilcoxon nonparametric signed-rank test and Spearman's correlation coefficient rho (ρ), the response to the items in the test and retest was similar with significantly related similar mean scores, with statistical significance (p< 0.001), during domain-wise (Table 12) and item by item-wise comparison (Table 11). The (ρ) for the item by item and per-domain were positively and strongly related at a two-tailed significance level of <0.001. All domains had significantly high (ρ) (>0.9), which indicated high reliability of the use of domain items across the two occasions in both questionnaires.

To complete the reliability analysis of the questionnaires, we performed further internal consistency analysis of the data by estimation of the Cronbach's alpha, inter-item correlation analysis, and the two-way mixed ICC (total agreement type), within a 95% confidence interval, and a two-tailed significance level of ≤0.05. Table 13 presents the internal consistency reliability analysis steps for both questionnaires in the test-retest evaluation. The overall alpha score for all domains was highly redundant for all items in both questionnaires, i.e., >0.9, which indicates excellent test-retest reliability, and excellent inter-rater reliability. The inter-item correlations and ICC for the items per domain in both questionnaires were >0.9, and indicated excellent internal reliability of the dimensions, and suggested a highly significant relationship between the questionnaires' domains.

We also calculated the inter-item correlations for each domain's items, intending to reach a corrected inter-item correlation mean of >0.3.

**Table 13:** Internal consistency analysis of the questionnaires in test-retest *The two-tailed significance level was less than 0.001 PCOSQoL: Polycystic Ovary Syndrome Quality of Life; SD: standard deviation

Questionnaire	Domain	Respondents to all items, n (%)	Cronbach's alpha	Inter-item correlation means	Intraclass correlation coefficient	95% confidence interval	
						Upper	Lower
PCOSQoL-45* (n=173)	A	116 (67.1)	.985	.972	.982	.969	.989
	B	137 (79.2)	.994	.989	.994	.992	.996
	C	114 (65.9)	.989	.978	.988	.981	.992
	D	159 (91.9)	.979	.960	.977	.968	.984
	E	145 (83.8)	.991	.986	.989	.982	.993
	Total	61 (35.3)	.996	.994	.996	.991	.998
PCOSQoL-53* N=195	A	172 (88.2)	.985	.973	.979	.936	.990
	B	122 (62.6)	.987	.986	.986	.980	.991
	C	163 (83.6)	.985	.978	.982	.965	.989
	D	162 (83.1)	.988	.980	.987	.982	.991
	E	139 (71.3)	.968	.942	.968	.955	.977
	Total	95 (48.7)	.991	.992	.984	.920	.994

We could not produce a heat map for the domains in both questionnaires because all domains showed a highly significant inter-item correlation coefficient of >0.9, i.e., showing an excellent strong correlation with each other with no preference. Internal consistency reliability analysis used Cronbach's alpha to aid item reduction. The item would be deleted if the domain's alpha coefficient scored more after its deletion.

Cronbach's alpha values before and after deletion for each item per domain are presented in Table 14. This step was considered as an item-reduction step through qualitative and quantitative analyses. All items were significantly related to each other, corresponding item with a two-tailed significance level of <0.001. We did not write it in the table for simplicity. This table included both the ICC coefficient and the internal consistency testing by Cronbach's alpha to test for the items that could be deleted to have a higher alpha score. Type A ICC used an absolute agreement definition.

**Table 14:** The application of Cronbach's alpha coefficient in item reduction for items in both PCOSQoL-45 and PCOSQoL-53 test-retest reliability analysis *These items would be deleted to increase the value of the Cronbach's alpha for its corresponding domains The two-tailed significance is >0.001 in all items and all domains PCOSQoL: Polycystic Ovary Syndrome Quality of Life

PCOSQoL-45							PCOSQoL-53					
Items	Test		Retest		Intraclass correlation coefficient		Items	Test		Retest		Intraclass correlation coefficient
	Cronbach's alpha if item deleted	Corrected item-total correlation	Cronbach's alpha if item deleted	Corrected item-total correlation				Cronbach's alpha if item deleted	Corrected item-total correlation	Cronbach's alpha if item deleted	Corrected item-total correlation	
A1	.869	.505	.797	.491	.944		A1	.816	.540	.773	.484	.973
A2	.863	.581	.801	.453	.944		A2	.806	.636	.768	.521	.941
A3	.859	.625	.800	.465	.916		A3	.811	.575	.765	.539	.930
A4	.842	.791	.775	.655	.945		A4	.803	.643	.757	.595	.993
A5	.847	.739	.778	.629	.925		A5	.809	.595	.760	.577	.961
A6	.855	.664	.786	.584	.970		A6	.815	.531	.770	.506	.965
A7*	.876	.386	.818	.265	.940		A7	.825	.427	.786	.359	.948
A8	.852	.694	.780	.619	.897		A8	.816	.521	.778	.442	.955
A9	.870	.504	.807	.402	.948		A9	.817	.514	.783	.393	.934
							A10*	.842	.256	.802	.217	.940
B1	.811	.394	.806	.378	.983							
B2	.790	.535	.781	.537	.981		B1	.946	.688	.915	.574	.958
B3	.771	.635	.771	.597	.963		B2	.942	.801	.903	.791	.978
B4	.785	.561	.777	.564	.963		B3	.948	.611	.914	.587	.956
B5	.772	.630	.757	.664	.975		B4	.945	.703	.914	.578	.957
B6	.784	.570	.775	.571	.950		B5	.937	.906	.904	.778	.950
B7	.793	.518	.793	.468	.984		B6	.940	.834	.904	.777	.966
							B7	.940	.833	.905	.756	.951
C1	.870	.630	.827	.408	.942		B8	.938	.900	.903	.818	.959
C2	.869	.611	.815	.532	.950		B9*	.952	.463	.921	.368	.925
C3	.862	.694	.802	.652	.941		B10	.942	.802	.908	.726	.947
C4	.867	.632	.809	.583	.927		B11	.939	.867	.909	.688	.919
C5	.858	.737	.800	.667	.951							
C6	.859	.728	.806	.614	.972		C1	.909	.623	.860	.421	.899
C7	.859	.727	.787	.761	.982		C2	.911	.565	.856	.488	.877
C8*	.883	.440	.832	.377	.983		C3	.907	.663	.848	.598	.970
C9*	.880	.482	.839	285	.934		C4	.907	.670	.853	.536	.934
							C5	.908	.640	.852	.547	.945
D1	.784	.424	.730	.371	.935		C6	.904	.716	.847	.607	.969
D2	.784	.419	.722	.438	.935		C7	.900	.782	.841	.680	.470
D3	.781	.441	.725	.408	.964		C8	.907	.667	.844	.651	.970
D4	.779	.462	.720	.437	.973		C9	.903	.741	.848	.596	.936
D5	.785	.406	.747	.246	.894		C10	.908	.646	.850	.573	.937
D6	.776	.504	.711	.524	.920		C11	.907	.663	.860	.421	.875
D7	.777	.483	.721	.436	.953							
D8	.776	.501	.724	.414	.946		D1	.874	.599	.790	.416	.945
D9	.779	.456	.733	.342	.971		D2	.883	.401	.797	.328	.957
D10	.775	.507	.718	.450	.965		D3	.880	.485	.797	.330	.923
D11	.780	.451	.735	.323	.886		D4	.872	.614	.789	.420	.914
							D5	.865	.708	.767	.610	.956
E1	.924	.726	.874	.606	.928		D6	.873	.590	.790	.413	.979
E2	.918	.808	.867	.689	.907		D7	.870	.658	.777	.557	.937
E3	.922	.758	.868	.669	.937		D8	.867	.685	.775	.561	.973
E4	.921	.778	.865	.714	.959		D9	.877	.575	.786	.485	.947
E5	.922	.748	.872	.631	.981		D10	.866	.696	.770	.591	.953
E6	.922	.751	.866	.694	.987		D11	.871	.627	.793	.411	.917
E7	.925	.699	.882	.489	.921							
E8	.928	.646	.881	.508	.948		E1*	.534	-.066-	.437	-.094-	.921
E9	.920	.794	.866	.708	.970		E2*	.528	.020	.430	-.021-	.965
							E3	.489	.183	.389	.112	.946
							E4*	.526	.042	.426	.008	.958
							E5	.482	.211	.389	.111	.957
							E6*	.482	.220	.405	.044	.938
							E7	.403	.406	.314	.272	.940
							E8	.446	.305	.280	.326	.966
							E9	.478	.221	.353	.209	.949
							E10	.382	.445	.244	.390	.976

For PCOSQoL-45, items A7 (Felt easily tired?) and C8 (Felt concerned about being overweight?) were deleted because Cronbach's alpha of the domain would be higher if deleted in the test analysis, which was confirmed in the retest analysis also. Item C9 (Felt concerned about a fast return to your previous weight after any weight loss?) was deleted because the Cronbach's alpha of the corresponding domain would increase if we deleted it. This was achieved in retest analysis only. All deleted items had the lowest corrected item-total correlation coefficients. On the other hand, the corrected item-total correlation coefficients values for the hair disorders and acne domain were lower than other domains in the questionnaire, especially for the items D1, D5, D9, and D11, but this did not affect the reliability of the domain to measure the respected outcome, given its high Cronbach's alpha and ICC coefficients. We recoded the PCOSQoL-45 to be PCOSQoL-42 to represent the resultant 42 items at their different domains after deleting the three items during the reliability analysis.

For PCOSQoL-53, five items were deleted to increase the Cronbach's alpha reliability of their corresponding domains. These items were A10 (Felt disappointed about the cure?), B9 (Felt dyspareunia during sexual intercourse?), E1 (Felt concerned about being overweight?), E2 (Felt the need to decrease your weight to control PCOS?), and E4 (Concerned to reduce your weight to be more attractive for your husband?). Item E6 (Felt concerned about menstruation at long intervals?) was deleted because the Cronbach's alpha of the corresponding domain would increase if we deleted it. This was achieved in the retest analysis only.

All deleted items had the lowest corrected item-total correlation coefficients. Item C7 (Embarrassed to engage in social activities because of your appearance?) had the lowest ICC coefficient. Still, it was not deleted as it contributed to the higher Cronbach's alpha of its corresponding domain. We recoded the PCOSQoL-53 to be PCOSQoL-47 to represent the resultant 47 items at their different domains after the deletion of the six items during the reliability analysis.

The two questionnaires were proven to have a significant-excellent level of reliability during the test and retest analysis; we can now proceed to use them in the field to examine their validity in different groups of women with PCOS. Subsequently, third resultant English and Arabic drafts (Tables 15, 16; Tables 17, 18 in the Appendix section) were created. These drafts will be discussed in a different article.

**Table 15:** Third draft for unmarried women with PCOS (PCOSQoL-42) PCOSQoL: Polycystic Ovary Syndrome Quality of Life

Domain	Code	Items	Never	Seldom	Quite often	Very often	Always
Psychological and emotional status	A1	Suffered from bad mood due to PCOS?	5	4	3	2	1
	A6	Felt pessimistic about the treatment?	5	4	3	2	1
	A9	Felt the urge to abandon treatments because of repetitive visits to doctors?	5	4	3	2	1
	A2	Felt frequent tantrums due to PCOS?	5	4	3	2	1
	A8	Experienced fear of diseases such as diabetes, hypertension, and heart disease?	5	4	3	2	1
	A3	Experienced trouble dealing with others?	5	4	3	2	1
	A5	Suffered from low self-esteem due to PCOS?	5	4	3	2	1
	A4	Blamed yourself for having PCOS?	5	4	3	2	1
Menstrual irregularities and fertility	B7	Felt the need to decrease your weight to control PCOS?	5	4	3	2	1
	B4	Felt concerned about future infertility?	5	4	3	2	1
	B2	Felt concerned about the complete cessation of menstruation?	5	4	3	2	1
	B1	Felt concerned about menstruation at long intervals?	5	4	3	2	1
	B3	Felt the regular need for oral contraceptive pills to control PCOS?	5	4	3	2	1
	B5	Felt you would accept all other PCOS manifestations if assured of pregnancy?	5	4	3	2	1
	B6	Experienced feelings of fear of cancer due to PCOS?	5	4	3	2	1
Body image	C1	Dissatisfied with some aspects of your appearance?	5	4	3	2	1
	C6	Tried to hide some flaws in your appearance?	5	4	3	2	1
	C2	Spent a significant amount of time checking your appearance in the mirror?	5	4	3	2	1
	C7	Ashamed of some part of your body?	5	4	3	2	1
	C5	Feared that others will discover flaws in your appearance?	5	4	3	2	1
	C4	Felt that others are speaking negatively about your appearance?	5	4	3	2	1
	C3	Avoided looking at your appearance in the mirror?	5	4	3	2	1
Hair disorders and acne	D1	Felt embarrassed about having excess facial and body hair?	5	4	3	2	1
	D6	Felt that alopecia is affecting your appearance?	5	4	3	2	1
	D2	Felt concerned about the progression pattern of excess body and facial hair?	5	4	3	2	1
	D7	Felt that alopecia led to a decrease in your attraction and femininity?	5	4	3	2	1
	D3	Felt concerned about rapid re-growth of unwanted hair after its removal?	5	4	3	2	1
	D8	Always wore a headscarf to cover your hair due to alopecia?	5	4	3	2	1
	D11	Feared that facial acne will leave permanent scars?	5	4	3	2	1
	D5	Felt that acne is affecting your appearance?	5	4	3	2	1
	D9	Felt that treatment of alopecia needs a long time and is worthless?	5	4	3	2	1
	D4	Felt the need to cover your body and face because of excess hair?	5	4	3	2	1
	D10	Avoided social circumstances due to alopecia?	5	4	3	2	1
Coping	E9	Tried to consult a medical expert about what you think is a flaw in your appearance?	5	4	3	2	1
	E7	Embarrassed to engage in social activities because of your appearance?	5	4	3	2	1
	E8	Compare your appearance with other women who you think are more physically attractive than you?	5	4	3	2	1
	E5	Felt disappointed about the cure?	5	4	3	2	1
	E6	Avoidance of social circumstances due to excess body hair?	5	4	3	2	1
	E1	Felt a lack of family support and acceptance of your disease?	5	4	3	2	1
	E4	Felt difficulty in communicating with other women who have PCOS?	5	4	3	2	1
	E2	Felt a lack of satisfaction with being a woman?	5	4	3	2	1
	E3	Concerned about your future role as a wife?	5	4	3	2	1

**Table 16:** Third draft for married women with PCOS (PCOSQoL-47) PCOSQoL: Polycystic Ovary Syndrome Quality of Life

Domain	Code	Items	Never	Seldom	Quite often	Very often	Always
Psychological and emotional status	A1	Suffered from bad mood due to PCOS?	5	4	3	2	1
	A7	Felt easily tired?	5	4	3	2	1
	A6	Felt pessimistic about the treatment?	5	4	3	2	1
	A9	Felt the urge to abandon treatments because of repetitive visits to doctors?	5	4	3	2	1
	A2	Felt frequent tantrums due to PCOS?	5	4	3	2	1
	A3	Experienced trouble dealing with others?	5	4	3	2	1
	A4	Blamed yourself for having PCOS?	5	4	3	2	1
	A8	Experienced fear of diseases such as diabetes, hypertension, and heart disease?	5	4	3	2	1
	A5	Suffered from low self-esteem due to PCOS?	5	4	3	2	1
Fertility and sexual life	B4	Felt fear of abortion?	5	4	3	2	1
	B6	Felt useless regarding sexual intercourse due to infertility?	5	4	3	2	1
	B2	Felt sad seeing pregnant women?	5	4	3	2	1
	B3	Experienced concern about future infertility?	5	4	3	2	1
	B5	Experienced fear of divorce or separation?	5	4	3	2	1
	B1	Felt sad seeing children?	5	4	3	2	1
	B8	Felt a lack of sexual desire?	5	4	3	2	1
	B11	Felt ashamed of sexual coldness/unresponsiveness?	5	4	3	2	1
	B7	Felt unsatisfied with sexual life?	5	4	3	2	1
	B10	Experienced a lack of orgasm?	5	4	3	2	1
Body image	C10	Tried to consult a medical expert about what you think is a flaw in your appearance?	5	4	3	2	1
	C1	Dissatisfied with some aspects of your appearance?	5	4	3	2	1
	C6	Tried to hide some flaws in your appearance?	5	4	3	2	1
	C11	Experienced fear of treatment complications?	5	4	3	2	1
	C8	Ashamed of some part of your body?	5	4	3	2	1
	C2	Spent a significant amount of time checking your appearance in the mirror?	5	4	3	2	1
	C7	Embarrassed to engage in social activities because of your appearance?	5	4	3	2	1
	C4	Felt that others are speaking negatively about your appearance?	5	4	3	2	1
	C9	Compare your appearance with other women who you think are more physically attractive than you?	5	4	3	2	1
	C5	Feared that others will discover flaws in your appearance?	5	4	3	2	1
	C3	Avoided looking at your appearance in the mirror?	5	4	3	2	1
Hair disorders and acne	D3	Felt concerned about rapid re-growth of unwanted hair after its removal?	5	4	3	2	1
	D2	Felt concerned about the progression pattern of excess body and facial hair?	5	4	3	2	1
	D6	Felt that acne is affecting your appearance?	5	4	3	2	1
	D1	Felt embarrassed about having excess facial and body hair?	5	4	3	2	1
	D8	Felt that alopecia led to a decrease in your attraction and femininity?	5	4	3	2	1
	D7	Felt that alopecia is affecting your appearance?	5	4	3	2	1
	D4	Felt the need to cover your body and face because of excess hair?	5	4	3	2	1
	D10	Felt that treatment of alopecia needs a long time and is worthless?	5	4	3	2	1
	D11	Feared that facial acne will leave permanent scars?	5	4	3	2	1
	D5	Avoidance of social circumstances due to excess body hair?	5	4	3	2	1
	D9	Always wore a headscarf or a veil to cover your hair due to alopecia?	5	4	3	2	1
Obesity and menstrual disorders	E3	Felt concerned about a fast return to your previous weight after any weight loss?	5	4	3	2	1
	E5	Felt concerned about the complete cessation of menstruation?	5	4	3	2	1
	E7	Felt the regular need for oral contraceptive pills to control PCOS?	5	4	3	2	1
	E9	Felt you would accept all other PCOS manifestations if assured of pregnancy?	5	4	3	2	1
	E8	Experienced feelings of fear of cancer due to PCOS?	5	4	3	2	1
	E10	Felt a lack of satisfaction with your current role as a wife?	5	4	3	2	1

## Discussion

The spectrum of complaints of women with PCOS is broad and contributes to PCOS phenotypic heterogeneity [1,12], marked psychological and emotional distress, and self-esteem misperception [2], which in turn negatively impacts their HRQoL due to the failure to conform to the idealized feminine aesthetic norms and optimal health standards [1,2].

The two most important questionnaires for HRQoL estimation in women with PCOS have been the 26-item PCOS questionnaire (PCOSQ) by Cronin et al. [15] and the most recent PCOSQ-50 by Nasiri-Amiri et al. [16]. The PCOSQ-50 is a 50-item questionnaire for women with PCOS, regardless of their marital status, with some culturally related modifications to suit the Iranian community [16], which is similar in its marital relations, traditions, and norms to the Iraqi community. These two reliable and validated questionnaires were the inspiration and the foundation for our work in developing the first disease-specific questionnaires for Arabic women with PCOS.

In this study, we tried to develop two HRQoL questionnaires, one for unmarried women and the second for married women with PCOS, in which we kept in mind that unmarried women in our conservative community are not sexually active, and the sexual activity is confined to married women; that is why we did not include sexual activity items in the questionnaire for unmarried women.

We used sophisticated qualitative and quantitative analyses for reliability and to create these two questionnaires for women with PCOS, with particular emphasis on the sexual and fertility issues faced frequently by these women. The qualitative and quantitative approaches started earlier at the item pool formation. The items development process lasted about four months, after which we got the final number of well-validated item pools in terms of content and face validity, by adopting the rules of Lawshe [8] and Ayre and Scally [9], to reach the maximally validated scores. We chose the minimal CVI and CVR as ≥0.65 and ≥0.3, respectively, as they corresponded to the 40-item pool concept, which is the maximal number described by the latter [9]. Although we set these two low cut-points as indicators, the minimal CVI and CVR for a given item in our scales were higher, i.e., >0.7.

The choice of having five domains for each questionnaire was determined by the redundancy of the items as frequently encountered by women with PCOS. The retrieved items in both questionnaires covered most of the PCOS aspects as creating a negative impact on HRQoL. We tried to include as many sexuality-related, acne, and hirsutism items as possible, to overcome some of the limitations of PCOSQ and PCOSQ-50.

The CVI and CVR of both questionnaires were around 0.92, which was similar to the 0.92 of the PCOSQ-50 [16], i.e., similar content validity indices. Other parameters were slightly in favor of our questionnaires like the higher rho (ρ) coefficient in test-retest reliability analysis (0.98 for PCOSQoL-42, range 0.94-0.96; and 0.96 for PCOSQoL-47, range 0.90-0.96) vs. 0.75 for PCOSQ-50. Cronbach's alpha for PCOSQoL-42 and PCOSQoL-47 was 0.996 and 0.991, respectively, vs. 0.88 for PCOSQ-50. This indicated that both questionnaires had higher reliability and internal consistency than PCOSQ-50.

We did not compare PCOSQoL-42 or PCOSQoL-47 to the 26-items PCOSQ of Cronin et al. because of its limited sensitivity, poor content and face validity, and reflectivity of HRQoL in these women with different phenotypes of PCOS [15,17], pertaining to physical symptoms and missing many QoL issues raised by the qualitative literature, such as acne [17].

The negative effect of PCOS on the psychological domains is evident and well-studied. Women with PCOS experience more incidences of neurotic disorders, aggression, and avoidance behavior [2,16,18]. The broad symptomatic spectrum of women with PCOS may affect their sexual function directly, or indirectly by obesity and body image dissatisfaction, which is frequently perceived by women with PCOS due to the associated clinical hyperandrogenic signs like hirsutism, female pattern hair loss, and acne [2,19]. The psychological and sexual domains are interrelated with causal relationships [19].

According to Nasiri-Amiri et al., the ability to tolerate the scope of the symptoms and signs of PCOS is affected by all the health dimensions (physical, mental, emotional, cognitive, social, and even the role functioning dimensions), and evaluating any coping mechanism is pivotal in estimating the HRQoL in any questionnaire [2,16,18].

There were two items related to the acne's effect on the general appearance that were kept in mind during the whole process of questionnaire evaluation. Acne is an important sign, which was frequently overlooked by the previous questionnaires [15,16]. The consideration of acne as a reliable sign of clinical hyperandrogenism in PCOS is controversial, given the previous observations among many women with different acne severities who had an average androgen level, and the lack of significant direct or causal correlation between hyperandrogenemia and acne. The underlying mechanism for this discrepancy might be attributed to the selective sebum production by the pilosebaceous unit in response to different concentrations of local or peripheral androgen in genetically susceptible women with PCOS [20].

The questionnaires' conceptual models were assessed through a psychometric cascade for reliability and validation to assess the questionnaire’s framework acceptability, responsiveness, and interpretability in a specific population [11]. There was no consensus on the scale characteristics to measure a requested health-related outcome from the patient perspective, especially scale length, item wordiness, or item length [21]. We tried our best to include positive, negative, and mixed wording formats in various items in our scales. This altering response alternative orientation was useful to restrict acquiescence by participants, without altering the scale [22].

Regarding the scale length, we used the 5-point Likert scale in both questionnaires, which was not short or long in scale length, by which the respondents were able to differentiate the intensity and directionality of their responses, with a neutral response in between. Increasing the scale length may have caused the participants to confuse how to translate their opinion into the scale, especially if they were cognitively apathetic or unprepared, leading to chances of resorting to heuristic responses [23].

The response time in the pilot study, the test-retest reliability analysis, and the final step of construct validity evaluation ranged from 15 to 30 minutes for both questionnaires, which is similar to what was primarily proposed by the research group during the item pool formation. Response time elapsed in answering questions is an important variable in the measuring scales, which may provide understanding related to some cognitive points during responding. It can be considered as an indicator of the recall ability that influences responding, for which any latency may indicate faulty design characteristics of the scale [24].

For test-retest reliability analysis, the timeline between the two occasions (five to seven days) was not so short, which helped to avoid recall or reporting bias among the participants that would have led them to memorize their previous responses in the initial test, and not so long during which the health status of the woman might change, as shown in previous reports [13].

The response rate was calculated for respondents who completed all items in a domain with no omission of any items (Table 10). The response rate of married women to their measuring scale showed no change between the two occasions as per the domain-wise and overall scale-wise patterns. While the response rate of unmarried women was profoundly low domain-wise and overall scale-wise patterns, we have no explanation for this discrepancy in response, given the same venue, interviewer, and logistics on both occasions. Still, the response rate ranged from acceptable to excellent in the two sets.

Our two questionnaires showed higher internal consistency, stability, and ICC during the test-retest reliability analysis compared to those of the PCOSQ-50 and PCOSQ [15,16]. Our questionnaire contained elements that had been overlooked or neglected by other questionnaires, a special domain for hair disorders and acne. A detailed sexual function and fertility domain may lead to higher reliability in assessing PCOS-related issues.

Hulin et al. had voiced concerns of a very high Cronbach's alpha coefficient during the reliability analysis, which may result from decreased validity due to acquiescence bias, especially in agreement responses. This type of bias, which was mostly seen in nonmotivated women, could not be neglected [22,25]. This was not the case in our questionnaires because there was a fluctuation in the responses and mean scores per domain, which ultimately explained the different item responses.

Our study's main strength lay in the study design, which included sequential analysis by both qualitative and quantitative statistical methods to reach the final scales for assessing the QoL in both married and unmarried women. During the whole study, we came across all PCOS phenotypes in different women, which provided an element of diversity in the responses for different subscales, and made the scales useful in any woman with PCOS regardless of the phenotype. The sexuality items were not selected for unmarried women, which might explain the high response rate across the study. However, the same items were a limiting factor for the married women's responses.

We used simple Arabic language with minimal use of ambiguous and medical terms to make the items more accessible to women from both groups. We also avoided words that might have various meanings and stuck to the use of original, one-meaning words.

Additionally, the PCOSQoL-42 and PCOSQoL-47 have addressed the concept of being sexually active and inactive properly by avoiding the inclusion of sexual themes in the scale described for unmarried women, which makes these two questionnaires applicable for any community with similar sexual norms. The structure of both scales provided the luxury of choosing between the two according to the sexual activity.

Due to its simple language format, these questionnaires can be translated to any language with ease and can be especially useful for communities similar to ours. Still, the measurement properties alone are not indicative of suitability among different populations [11].

## Conclusions

Both PCOSQoL-47 and PCOSQoL-42 are simple, highly reliable, easily comprehensible, and self-administered conceptual tools to evaluate different HRQoL measures in married and unmarried women with PCOS, whose first or native language is Arabic, regardless of their phenotype. After translation, these questionnaires could facilitate further studies in Arabic-speaking communities and other communities with similar norms regarding marriage and sexuality.

## Appendices

**Table 17:** The Arabic version of PCOSQoL-42 for unmarried women with PCOS PCOSQoL: Polycystic Ovary Syndrome Quality of Life

	الرمز	الفقرات	ابدا	نادرا	غالبا	غالبا جدا	دائما
حقل الحالة النفسية و العاطفية	A1	عانيت من مزاج سيء بسبب تكيس المبايض؟	5	4	3	2	1
	A6	شعرت بالتشاؤم حول العلاج؟	5	4	3	2	1
	A9	شعرت برغبة لترك العلاج بسبب الزيارات المتكررة للأطباء؟	5	4	3	2	1
	A2	انتابتك نوبات غضب متكررة بسبب تكيس المبايض؟	5	4	3	2	1
	A8	احسست بالخوف من الامراض مثل السكري, ارتفاع ضغط الدم, و امراض القلب؟	5	4	3	2	1
	A3	واجهت مشكلة في التعامل مع الاخرين؟	5	4	3	2	1
	A5	عانيت من تدني احترام الذات بسبب تكيس المبايض	5	4	3	2	1
	A4	لمت نفسك على وجود تكيس المبايض؟	5	4	3	2	1
حقل اضطرابات الدورة الشهرية و الخصوبة	B7	شعرت بالحاجة لتقليل وزنك للتحكم في تكيس المبايض؟	5	4	3	2	1
	B4	شعرت بالقلق إزاء عدم الانجاب مستقبلا؟	5	4	3	2	1
	B2	شعرت بالقلق حيال التوقف التام للدورة الشهرية؟	5	4	3	2	1
	B1	شعرت بالقلق حيال تباعد فترات الدورة الشهرية؟	5	4	3	2	1
	B3	شعرت بالحاجة الى استخدام حبوب منع الحمل للسيطرة على تكيس المبايض؟	5	4	3	2	1
	B5	قبلت بكل مظاهر تكيس المبيض الاخرى اذا حصلت على ضمان بالحمل؟	5	4	3	2	1
	B6	راودتك مشاعر الخوف من السرطان بسبب تكيس المبايض؟	5	4	3	2	1
حقل صورة الجسم	C1	كنت غير راضية عن بعض جوانب مظهرك؟	5	4	3	2	1
	C6	حاولت اخفاء بعض العيوب في مظهرك؟	5	4	3	2	1
	C2	قضيت الكثير من الوقت في التحقق من مظهرك امام المرأة؟	5	4	3	2	1
	C7	خجلت من بعض اجزاء جسمك؟	5	4	3	2	1
	C5	خشيت ان يكتشف الاخرون عيوبا في مظهرك؟	5	4	3	2	1
	C4	شعرت ان الاخرين يتحدثون سلبيا عن مظهرك؟	5	4	3	2	1
	C3	تحاشيت النظر الى مظهرك في المرأة؟	5	4	3	2	1
حقل اضطرابات الشعر و حب الشباب	D1	شعرت بالحرج بسبب شعر الوجه و الجسم الزائد؟	5	4	3	2	1
	D6	شعرت ان تساقط الشعر الكثيف يشوه مظهرك؟	5	4	3	2	1
	D2	شعرت بالقلق من نمط ازدياد نمو الشعر في الوجه و الجسم؟	5	4	3	2	1
	D7	شعرت ان تساقط الشعر الكثيف يقلل من جاذبيتك و انوثتك؟	5	4	3	2	1
	D3	شعرت بالقلق ازاء النمو السريع للشعر غير المرغوب فيه بعد ازالته؟	5	4	3	2	1
	D8	ارتديت وشاح الراس او الحجاب لتغطية شعرك بسبب تساقط الشعر الكثيف؟	5	4	3	2	1
	D11	خشيت ان يترك حب الشباب ندبا دائمية؟	5	4	3	2	1
	D5	شعرت ان حب الشباب يشوه مظهرك؟	5	4	3	2	1
	D9	شعرت ان علاج تساقط الشعر يحتاج لفترة طويلة و انه غير مجد؟	5	4	3	2	1
	D4	شعرت بحاجة لتغطية جسمك و وجهك بسبب الشعر الزائد؟	5	4	3	2	1
	D10	تجنبت المناسبات الاجتماعية بسبب تساقط الشعر؟	5	4	3	2	1
حقل المواجهة	E9	حاولت الحصول على استشارة طبية بشأن ما تعتقدين انه عيب في مظهرك؟	5	4	3	2	1
	E7	شعرت بالحرج من الظهور في المناسبات الاجتماعية بسبب مظهرك؟	5	4	3	2	1
	E8	قارنت مظهرك مع نساء أخريات ممن تعتقدين انهن يمتلكن مواصفات جسمانية اكثر جاذبية منك؟	5	4	3	2	1
	E5	شعرت بخيبة الامل حيال الشفاء؟	5	4	3	2	1
	E6	تجنبت المناسبات الاجتماعية بسبب زيادة شعر الجسم؟	5	4	3	2	1
	E1	احسست بفقدان الدعم الاسري لقبول حالتك المرضية؟	5	4	3	2	1
	E4	شعرت بصعوبة في التواصل مع نساء اخريات لديهن تكيس المبايض؟	5	4	3	2	1
	E2	شعرت بعدم الرضا عن كونك امرأة؟	5	4	3	2	1
	E3	شعرت بالقلق إزاء دورك المستقبلي كزوجة؟	5	4	3	2	1

**Table 18:** The Arabic version of PCOSQoL-47 for married women with PCOS PCOSQoL: Polycystic Ovary Syndrome Quality of Life

	الرمز		ابدا	نادرا	غالبا	غالبا جدا	دائما
حقل الحالة النفسية و العاطفية	A1	عانيت من مزاج سيء بسبب تكيس المبايض؟	5	4	3	2	1
	A7	احسست بالإرهاق بسهولة؟	5	4	3	2	1
	A6	شعرت بالتشاؤم حول العلاج؟	5	4	3	2	1
	A9	شعرت برغبة لترك العلاج بسبب الزيارات المتكررة للأطباء؟	5	4	3	2	1
	A2	انتابتك نوبات غضب متكررة بسبب تكيس المبايض؟	5	4	3	2	1
	A3	واجهت مشكلة في التعامل مع الاخرين؟	5	4	3	2	1
	A4	لمت نفسك على وجود تكيس المبايض؟	5	4	3	2	1
	A8	احسست بالخوف من الامراض مثل السكري, ارتفاع ضغط الدم, و امراض القلب؟	5	4	3	2	1
	A5	عانيت من تدني احترام الذات بسبب تكيس المبايض	5	4	3	2	1
حقل الخصوبة و الحياة الجنسية	B4	خشيت الاجهاض؟	5	4	3	2	1
	B6	شعرت بعدم جدوى الاتصال الجنسي بسبب العقم؟	5	4	3	2	1
	B2	شعرت بالحزن عند رؤيتك للنساء الحوامل؟	5	4	3	2	1
	B3	شعرت بالقلق إزاء عدم الانجاب مستقبلا؟	5	4	3	2	1
	B5	خشيت الطلاق او الانفصال؟	5	4	3	2	1
	B1	شعرت بالحزن عند رؤيتك الاطفال؟	5	4	3	2	1
	B8	شعرت بنقص الرغبة الجنسية؟	5	4	3	2	1
	B11	شعرت بالخجل من البرود الجنسي و عدم الاستجابة؟	5	4	3	2	1
	B7	شعرت بعدم الرضا عن حياتك الجنسية؟	5	4	3	2	1
	B10	شعرت بفقدان الرعشة الجنسية؟	5	4	3	2	1
حقل صورة الجسم	C10	حاولت الحصول على استشارة طبية بشأن ما تعتقدين انه عيب في مظهرك؟	5	4	3	2	1
	C1	كنت غير راضية عن بعض جوانب مظهرك؟	5	4	3	2	1
	C6	حاولت اخفاء بعض العيوب في مظهرك؟	5	4	3	2	1
	C11	قلقت من المضاعفات المترتبة على العلاج؟	5	4	3	2	1
	C8	خجلت من بعض اجزاء جسمك؟	5	4	3	2	1
	C2	قضيت الكثير من الوقت في التحقق من مظهرك امام المرأة؟	5	4	3	2	1
	C7	شعرت بالحرج من الظهور في المناسبات الاجتماعية بسبب مظهرك؟	5	4	3	2	1
	C4	شعرت ان الاخرين يتحدثون سلبيا عن مظهرك؟	5	4	3	2	1
	C9	قارنت مظهرك مع نساء أخريات ممن تعتقدين انهن يمتلكن مواصفات جسمانية اكثر جاذبية منك؟؟	5	4	3	2	1
	C5	خشيت ان يكتشف الاخرون عيوبا في مظهرك؟	5	4	3	2	1
	C3	تحاشيت النظر الى مظهرك في المرأة؟	5	4	3	2	1
حقل اضطرابات الشعر و حب الشباب	D3	شعرت بالقلق ازاء النمو السريع للشعر غير المرغوب فيه بعد ازالته؟	5	4	3	2	1
	D2	شعرت بالقلق من نمط ازدياد نمو الشعر في الوجه و الجسم؟	5	4	3	2	1
	D6	شعرت ان حب الشباب يشوه مظهرك؟	5	4	3	2	1
	D1	شعرت بالحرج بسبب شعر الوجه و الجسم الزائد؟	5	4	3	2	1
	D8	شعرت ان تساقط الشعر الكثيف يقلل من جاذبيتك و انوثتك؟	5	4	3	2	1
	D7	شعرت ان تساقط الشعر الكثيف يشوه مظهرك؟	5	4	3	2	1
	D4	شعرت بحاجة لتغطية جسمك و وجهك بسبب الشعر الزائد؟	5	4	3	2	1
	D10	شعرت ان علاج تساقط الشعر يحتاج لفترة طويلة و انه غير مجد؟	5	4	3	2	1
	D11	خشيت ان يترك حب الشباب ندبا دائمية؟	5	4	3	2	1
	D5	تجنبت المناسبات الاجتماعية بسبب زيادة شعر الجسم؟	5	4	3	2	1
	D9	ارتديت وشاح الرأس او الحجاب لتغطية شعرك بسبب تساقط الشعر الكثيف؟	5	4	3	2	1
حقل السمنة و اضطرابات الدورة الشهرية	E3	شعرت بالقلق حيال العودة السريعة الى وزنك السابق بعد اي فقدان للوزن؟	5	4	3	2	1
	E5	شعرت بالقلق حيال التوقف التام للدورة الشهرية؟	5	4	3	2	1
	E7	شعرت بالحاجة الى استخدام حبوب منع الحمل للسيطرة على تكيس المبايض؟	5	4	3	2	1
	E9	قبلت بكل مظاهر تكيس المبيض الاخرى اذا حصلت على ضمان بالحمل؟	5	4	3	2	1
	E8	راودتك مشاعر الخوف من السرطان بسبب تكيس المبايض؟	5	4	3	2	1
	E10	شعرت بعدم الرضا عن دورك الحالي كزوجة؟	5	4	3	2	1

A: Informed consent form for unmarried women with polycystic ovary syndrome in Basrah

·        Name of Principal Investigator: Samih Abed Odhaib

·        Name of Organization: Faiha Specialized Diabetes Endocrine and Metabolism Center

·        Name of Project: Development of the First Health-Related Quality of Life Questionnaires for Arabic Women with Polycystic Ovary Syndrome: Formation, Reliability Analysis, and Validation of the PCOSQoL-47 and PCOSQoL-42 Questionnaires

This Informed Consent Form has two parts:                                                                                

·        Information Sheet (to share information about the study with you).

·        Certificate of Consent (for signatures if you choose to participate)

You will be given a copy of the full Informed Consent Form

Part I: Information sheet

Introduction

We represent the study group of FDEMC. We are trying to validate a new questionnaire dealing with the health-related quality of life in sexually active women with polycystic ovary syndrome (PCOS). PCOS is the commonest female endocrinopathy. We want you to participate in this study to help us validate our new questionnaire that uses the Arabic language for the first time, which will be useful for all Arabic-speaking women. You are free to participate or not, and you have all the right to agree or not. You are not obliged to give us consent today, you have all the week-long to study the consent and give us the response in your next visit a week from now, during this period you are free to contact any one of the study group to ask about any aspect you found it peculiar and need discussion. Whatever your decision will be, it will not affect our level of medical services provided to you. Keep in mind that you can withdraw your consent at any time before the complete publication of the study, and you are not obliged to give us any reasons. Again this will not affect our medical decision regarding your management by any means. You may have no questions now, but during the study, you may have questions, feel free to inform us to answer them. Is this understandable to you?

Purpose of the research

PCOS is a common endocrinopathy affecting many women in their reproductive life and affecting their quality of life negatively at different levels. It affects the marital life, conception, childbearing, and body image, and may associate important diseases like hypertension, diabetes mellitus, and metabolic syndrome, in addition to its association with many psychological illnesses. We need to create and validate a questionnaire that may include most of your concerns about PCOS, that many women may consider important. We will try to measure the questionnaire reliability in our community in Basrah as an Arabic-speaking city. Is this understandable to you?

Type of Research Intervention

We will give you a questionnaire in Arabic language containing items concerned with your health-related quality of life and the effect of PCOS on different aspects of your life. The question will try to cover all aspects of your health related-quality of life. These questions were created and carefully selected and located in different domains with the simplest language terms to be self-explainable. You will have around 45 different questions in Arabic, you may read them carefully and answer them by selecting the appropriate scale of its effect on your life. These questions are about your feelings and sensations in the last two weeks. You will have all questions supplied with the same scale of the answer which is called (Likert scale), of five points. Point 1 represents the severe effect on your HRQoL, while point five represents the mildest effect on your HRQoL during that period. Please feel free to ask any of the workgroups about any question you may find ambiguous or difficult to understand. You are not obliged to answer any question you are not comfortable with it, and you are not obliged to give any justification to do that by anyone. And always remember that any answer will not affect our level of services that were planned for you by any means.

This questionnaire may take 12-25 minutes to be answered, you can take your time in a quiet place provided by the study group, where you calmly replied to the questions. You are free to seek help from a partner, or a family, or any of the workgroup, or you answer without help from anyone.

You (may) find another questionnaire attached to the first questionnaire, this will deal with the general aspect of the quality of life that was previously validated by the World Health Organization (WHO). It contains 26 questions in Arabic. Feel free to answer them or not, and you can ask about any question of them. This questionnaire may take 6-10 minutes.

We will use the second questionnaire to validate the first questionnaire through multiple statistical methods, to reach the final form of the questionnaire that represents the main complaints of women with PCOS, and their quality of life.

You are not restricted by any time, but the provider will register the exact timing that the questionnaires were provided to you, to calculate the time elapsed to answer the questions. You are free to leave any question or domain at any time during the questionnaire reading, and you will not be asked for any justification, and will never affect our judgment for your PCOS management. All the data and information provided by you in the questionnaire are confidential, and no one will know about it except the study group, and absolutely no sharing of data by any means will occur, now or later. Do you have any questions?

All the participants will be dealt with as (registration numbers), not by names during the study progress. To keep the data as secrete as possible. All information will be registered as numbers on our datasheet directly with no names, only the registration number at the FDEMC database. The whole study is screening for any effect of the PCOS on your HRQoL and not involve any invasive or noninvasive investigations by any means. Is this understandable to you?

Participant Selection

You are being invited to take part in this research because we feel that your experience with PCOS can contribute much to our understanding and knowledge of local health practices, and may help us to decrease the effect of this syndrome on the quality of life of women. Is this understandable to you?

Voluntary Participation

Your participation in this research is entirely voluntary. It is your choice whether to participate or not. If you choose not to participate, all the services you receive at FDEMC will continue and nothing will change.

If you decide not to take part in this research study, do you know what your options are? Do you know that you do not have to take part in this research study if you do not wish to? Do you have any questions?

You need to know:

·        We need to participate to help us to enlighten the effect of PCOS on the HRQoL of sexually inactive women (Unmarried), in Basrah first and then to use it in any Arabic-speaking city. You will not be asked about any sexuality issues.

·        You will not be subjected to any discussion group or focus group or any group therapy by any mean, and you will not share your information or data with anyone whether similar to your complaint or not, all the participants are blind to each other, but not to the study group.

·        We will never ask you about any person's beliefs, practices, or stories, that you are not comfortable with sharing.

·         All interviews will take place at FDEMC clinics, by the workgroups exclusively, not anyone else. All the interviews are verbal and not recorded by any means. Only data registration will take place on our datasheet by the first author (Samih Abed Odhaib) exclusively, without name identification, but only registration numbers at FDEMC. And he will be the only person that can access your data.

·        All the questionnaire papers will be kept by the first author with no names on them, only the registration number. All paper-packs will be destroyed by the first author on study completion after publication, with no hard copies to be kept.

Duration

The proposed time of the study is about 20 months, the study will end in August 2020. The data will be available for analysis after that date, and you will be free to participate in the study at any time, given you will fulfill the enrollment criteria at that time. On the other hand, if you accept to participate in the study you can withdraw your participation at any time before the publication of the whole study, i.e. August 2020 will be the deadline for the enrollment not for publication. Do you have any more questions?

Risks

We are asking you to share with us some very personal and confidential information, and you may feel uncomfortable talking about some of the topics. We already Omitted the sexuality items from your copy of the questionnaire. You do not have to answer any question or take part in the discussion/interview/questionnaire if you don't wish to do so, and that is also fine. You do not have to give us any reason for not responding to any question, or for refusing to take part in the interview or questionnaire.

Benefits

There will be no direct benefit to you, but your participation is likely to help us find out more about how to deal with PCOS's effect on the HRQoL in our community.

Reimbursements

You will not be provided any incentive to take part in the research. There will be no monetary reward to you by any kind.

Can you tell me if you have understood correctly the benefits that you will have if you take part in the study? Do you know if the study will pay for your travel costs and time lost, and do you know how much you will be reimbursed? Do you have any other questions?

Confidentiality

The research being done in the community may draw attention and if you participate, you may be asked questions by other people in the community. We will not be sharing information about you with anyone outside of the research team. The information that we collect from this research project will be kept private. Any information about you will have a number on it instead of your name. Only the researchers will know what your number is and we will lock that information up with a lock and key. It will not be shared with or given to anyone except the first author.

Sharing the Results

Nothing that you tell us today will be shared with anybody outside the research team, and nothing will be attributed to you by name. The knowledge that we get from this research will be shared with you before it is published. Each participant will receive a summary of the results in private.

Right to Refuse or Withdraw

You do not have to take part in this research if you do not wish to do so, and choosing to participate will not affect the medical services and your evaluations in any way. You may stop participating in them at any time that you wish without any effect on the level of medical care being affected.

Whom to Contact

If you have any questions, you can ask them now or later. If you wish to ask questions later, you may contact any of the following:

·        Samih Abed Odhaib MD. Phone: 009647816787885. Email: .

·        Mahmood Thamer Jallod Altemimi MD. Phone: 009647807326088. Email:.

·        Husam Jihad Imran MD: Phone: 009647721827666. Email:

This proposal has been reviewed and approved by FDEMC ethical committee, which is a committee whose task is to make sure that research participants are protected from harm. If you wish to find about more about it, contact the head of the committee:

Ibrahim Abbood Zaboon, MD, MSc (Endocrine), Adult Endocrinologist. Phone: 009647801230556, Email:

Do you know that you do not have to take part in this study if you do not wish to? You can say no if you wish to? Do you know that you can ask me questions later if you wish to? Do you know that I have given the contact details of the person who can give you more information about the study? You can ask me any more questions about any part of the research study if you wish to. Do you have any questions? 

Part II: Certificate of consent

I have read the foregoing information, or it has been read to me. I have had the opportunity to ask questions about it and any questions I have been asked have been answered to my satisfaction. I consent voluntarily to be a participant in this study

Print Name of Participant:

Signature of Participant:

Date: Day/month/year

If illiterate

I have witnessed the accurate reading of the consent form to the potential participant, and the individual has had the opportunity to ask questions. I confirm that the individual has given consent freely.

Print name of witness:                                                       Thumbprint of participant

Signature of witness:

Date: Day/month/year

Statement by the researcher/person taking consent

I have accurately read out the information sheet to the potential participant, and to the best of my ability made sure that the participant understands that the following will be done:

1. She understands the goal of the study and the different aspects of it.

2.She had all time to ask questions and was free to participate in the study or not.

3.She was aware that all her data are confidential.

I confirm that the participant was given an opportunity to ask questions about the study, and all the questions asked by the participant have been answered correctly and to the best of my ability. I confirm that the individual has not been coerced into giving consent, and the consent has been given freely and voluntarily.

A copy of this informed consent form has been provided to the participant.

Print Name of Researcher/person taking the consent:                         

Signature of Researcher/person taking the consent:

Date: Day/month/year                 

B: Informed consent form for married women with polycystic ovary syndrome in Basrah

·        Name of Principal Investigator: Samih Abed Odhaib

·        Name of Organization: Faiha Specialized Diabetes Endocrine and Metabolism Center

·        Name of Project: Development of the First Health-Related Quality of Life Questionnaires for Arabic Women with Polycystic Ovary Syndrome: Formation, Reliability Analysis, and Validation of the PCOSQoL-47 and PCOSQoL-42 Questionnaires

This Informed Consent Form has two parts:

·        Information Sheet (to share information about the study with you).

·        Certificate of Consent (for signatures if you choose to participate)

You will be given a copy of the full Informed Consent Form

Part I: Information sheet

Introduction

We represent the study group of FDEMC. We are trying to validate a new questionnaire dealing with the health-related quality of life in sexually active women with polycystic ovary syndrome (PCOS). PCOS is the commonest female endocrinopathy. We want you to participate in this study to help us validate our new questionnaire that uses the Arabic language for the first time, which will be useful for all Arabic-speaking women. You are free to participate or not, and you have all the right to agree or not. You are not obliged to give us consent today, you have all the week-long to study the consent and give us the response in your next visit a week from now, during this period you are free to contact any one of the study group to ask about any aspect you found it peculiar and need discussion. Whatever your decision will be, it will not affect our level of medical services provided to you. Keep in mind that you can withdraw your consent at any time before the complete publication of the study, and you are not obliged to give us any reasons. Again this will not affect our medical decision regarding your management by any means. You may have no questions now, but during the study, you may have questions, feel free to inform us to answer them. Is this understandable to you?

Purpose of the research

PCOS is a common endocrinopathy affecting many women in their reproductive life and affecting their quality of life negatively at different levels. It affects the marital life, conception, childbearing, and body image, and may associate important diseases like hypertension, diabetes mellitus, and metabolic syndrome, in addition to its association with many psychological illnesses. We need to create and validate a questionnaire that may include most of your concerns about PCOS, that many women may consider important. We will try to measure the questionnaire reliability in our community in Basrah as an Arabic-speaking city. Is this understandable to you?

Type of Research Intervention

We will give you a questionnaire in Arabic language containing items concerned with your health-related quality of life and the effect of PCOS on different aspects of your life. The question will try to cover all aspects of your health related-quality of life. These questions were created and carefully selected and located in different domains with the simplest language terms to be self-explainable. You will have around 45 different questions in Arabic, you may read them carefully and answer them by selecting the appropriate scale of its effect on your life. These questions are about your feelings and sensations in the last two weeks. You will have all questions supplied with the same scale of the answer which is called (Likert scale), of five points. Point 1 represents the severe effect on your HRQoL, while point five represents the mildest effect on your HRQoL during that period. Please feel free to ask any of the workgroups about any question you may find ambiguous or difficult to understand. You are not obliged to answer any question you are not comfortable with it, and you are not obliged to give any justification to do that by anyone. And always remember that any answer will not affect our level of services that were planned for you by any means.

This questionnaire may take 12-25 minutes to be answered, you can take your time in a quiet place provided by the study group, where you calmly replied to the questions. You are free to seek help from a partner, or a family, or any of the workgroup, or you answer without help from anyone.

You (may) find another questionnaire attached to the first questionnaire, this will deal with the general aspect of the quality of life that was previously validated by the World Health Organization (WHO). It contains 26 questions in Arabic. Feel free to answer them or not, and you can ask about any question of them. This questionnaire may take 6-10 minutes.

We will use the second questionnaire to validate the first questionnaire through multiple statistical methods, to reach the final form of the questionnaire that represents the main complaints of women with PCOS, and their quality of life.

You are not restricted by any time, but the provider will register the exact timing that the questionnaires were provided to you, to calculate the time elapsed to answer the questions. You are free to leave any question or domain at any time during the questionnaire reading, and you will not be asked for any justification, and will never affect our judgment for your PCOS management. All the data and information provided by you in the questionnaire are confidential, and no one will know about it except the study group, and absolutely no sharing of data by any means will occur, now or later. Do you have any questions?

All the participants will be dealt with as (registration numbers), not by names during the study progress. To keep the data as secrete as possible. All information will be registered as numbers on our datasheet directly with no names, only the registration number at the FDEMC database. The whole study is screening for any effect of the PCOS on your HRQoL and not involve any invasive or noninvasive investigations by any means. Is this understandable to you?

Participant Selection

You are being invited to take part in this research because we feel that your experience with PCOS can contribute much to our understanding and knowledge of local health practices, and may help us to decrease the effect of this syndrome on the quality of life of women. Is this understandable to you?

Voluntary Participation

Your participation in this research is entirely voluntary. It is your choice whether to participate or not. If you choose not to participate, all the services you receive at FDEMC will continue and nothing will change.

If you decide not to take part in this research study, do you know what your options are? Do you know that you do not have to take part in this research study if you do not wish to? Do you have any questions?

You need to know:

·        We need to participate to help us to enlighten the effect of PCOS on the HRQoL of sexually active women (Married), in Basrah first and then to use it in any Arabic-speaking city.

·        You will not be subjected to any discussion group or focus group or any group therapy by any mean, and you will not share your information or data with anyone whether similar to your complaint or not, all the participants are blind to each other, but not to the study group.

·        We will never ask you about any person's beliefs, practices, or stories, that you are not comfortable with sharing.

·         All interviews will take place at FDEMC clinics, by the workgroups exclusively, not anyone else. All the interviews are verbal and not recorded by any means. Only data registration will take place on our datasheet by the first author (Samih Abed Odhaib) exclusively, without name identification, but only registration numbers at FDEMC. And he will be the only person that can access your data.

·        All the questionnaire papers will be kept by the first author with no names on them, only the registration number. All papers will be destroyed by the first author on study completion after the proposed date of August, with no copies to be kept.

Duration

The proposed time of the study is about 20 months, the study will end in August 2020. The data will be available for analysis during that period, and you will be free to participate in the study at any time, given you will fulfill the enrollment criteria at that time. On the other hand, if you accept to participate in the study you can withdraw your participation at any time before the publication of the whole study, i.e. August 2020 will be the deadline for the enrollment not for publication. Do you have any more questions?

Risks

We are asking you to share with us some very personal and confidential information, and you may feel uncomfortable talking about some of the topics. You do not have to answer any question or take part in the discussion/interview/questionnaire if you don't wish to do so, and that is also fine. You do not have to give us any reason for not responding to any question, or for refusing to take part in the interview or questionnaire.

Benefits

There will be no direct benefit to you, but your participation is likely to help us find out more about how to deal with PCOS's effect on the HRQoL in our community.

Reimbursements

You will not be provided any incentive to take part in the research. There will be no monetary reward to you by any kind.

Can you tell me if you have understood correctly the benefits that you will have if you take part in the study? Do you know if the study will pay for your travel costs and time lost, and do you know how much you will be reimbursed? Do you have any other questions?

Confidentiality

The research being done in the community may draw attention and if you participate you may be asked questions by other people in the community. We will not be sharing information about you with anyone outside of the research team. The information that we collect from this research project will be kept private. Any information about you will have a number on it instead of your name. Only the researchers will know what your number is and we will lock that information up with a lock and key. It will not be shared with or given to anyone except the first author.

Sharing the Results

Nothing that you tell us today will be shared with anybody outside the research team, and nothing will be attributed to you by name. The knowledge that we get from this research will be shared with you before it is published. Each participant will receive a summary of the results in private.

Right to Refuse or Withdraw

You do not have to take part in this research if you do not wish to do so, and choosing to participate will not affect the medical services and your evaluations in any way. You may stop participating in them at any time that you wish without any effect on the level of medical care being affected.

Whom to Contact

If you have any questions, you can ask them now or later. If you wish to ask questions later, you may contact any of the following:

·        Samih Abed Odhaib MD. Phone: 009647816787885. Email: .

·        Mahmood Thamer Jallod Altemimi MD. Phone: 009647807326088. Email:.

·        Husam Jihad Imran MD: Phone: 009647721827666. Email:

This proposal has been reviewed and approved by FDEMC ethical committee, which is a committee whose task is to make sure that research participants are protected from harm. If you wish to find about more about it, contact the head of the committee:

Ibrahim Abbood Zaboon, MD, MSc (Endocrine), Adult Endocrinologist. Phone: 009647801230556, Email:

Do you know that you do not have to take part in this study if you do not wish to? You can say no if you wish to? Do you know that you can ask me questions later if you wish to? Do you know that I have given the contact details of the person who can give you more information about the study? You can ask me any more questions about any part of the research study if you wish to. Do you have any questions? 

Part II: Certificate of consent

I have read the foregoing information, or it has been read to me. I have had the opportunity to ask questions about it and any questions I have been asked have been answered to my satisfaction. I consent voluntarily to be a participant in this study

Print Name of Participant:

Signature of Participant:

Date: Day/month/year

If illiterate

I have witnessed the accurate reading of the consent form to the potential participant, and the individual has had the opportunity to ask questions. I confirm that the individual has given consent freely.

Print name of witness:                                                       Thumbprint of participant

Signature of witness:

Date: Day/month/year

Statement by the researcher/person taking consent

I have accurately read out the information sheet to the potential participant, and to the best of my ability made sure that the participant understands that the following will be done:

1. She understands the goal of the study and the different aspects of it.

2. She had all time to ask questions and was free to participate in the study or not.

3. She was aware that all her data are confidential.

I confirm that the participant was given an opportunity to ask questions about the study, and all the questions asked by the participant have been answered correctly and to the best of my ability. I confirm that the individual has not been coerced into giving consent, and the consent has been given freely and voluntarily.

A copy of this informed consent form has been provided to the participant.

Print Name of Researcher/person taking the consent:                         

Signature of Researcher/person taking the consent:

Date: Day/month/year:                
